# Targeting DRP1 with Mdivi-1 to correct mitochondrial abnormalities in ADOA+ syndrome

**DOI:** 10.1172/jci.insight.180582

**Published:** 2024-06-25

**Authors:** Yan Lin, Dongdong Wang, Busu Li, Jiayin Wang, Ling Xu, Xiaohan Sun, Kunqian Ji, Chuanzhu Yan, Fuchen Liu, Yuying Zhao

**Affiliations:** 1 Research Institute of Neuromuscular and Neurodegenerative Diseases and Department of Neurology, Qilu Hospital, Cheeloo College of Medicine, Shandong University, Jinan, Shandong, China.; 2 Mitochondrial Medicine Laboratory, Qilu Hospital (Qingdao), Shandong University, Qingdao, Shandong, China.; 3 Brain Science Research Institute, Shandong University, Jinan, Shandong, China.

**Keywords:** Neuroscience, Ophthalmology, Autophagy, Drug therapy, Mitochondria

## Abstract

Autosomal dominant optic atrophy plus (ADOA+) is characterized by primary optic nerve atrophy accompanied by a spectrum of degenerative neurological symptoms. Despite ongoing research, no effective treatments are currently available for this condition. Our study provided evidence for the pathogenicity of an unreported c.1780T>C variant in the OPA1 gene through patient-derived skin fibroblasts and an engineered HEK293T cell line with OPA1 downregulation. We demonstrate that OPA1 insufficiency promoted mitochondrial fragmentation and increased DRP1 expression, disrupting mitochondrial dynamics. Consequently, this disruption enhanced mitophagy and caused mitochondrial dysfunction, contributing to the ADOA+ phenotype. Notably, the Drp1 inhibitor, mitochondrial division inhibitor-1 (Mdivi-1), effectively mitigated the adverse effects of OPA1 impairment. These effects included reduced Drp1 phosphorylation, decreased mitochondrial fragmentation, and balanced mitophagy. Thus, we propose that intervening in DRP1 with Mdivi-1 could correct mitochondrial abnormalities, offering a promising therapeutic approach for managing ADOA+.

## Abstract 2

Dysfunction of an autosomal dominant optic atrophy plus–associated OPA1 can be corrected by Mdivi-1–mediated inhibition of Drp1.

## Introduction

Autosomal dominant optic atrophy (ADOA) (OMIM #165500) is a commonly inherited optic neuropathy, typically manifesting during adolescence (1). Currently, effective treatments for this condition are lacking. Most cases are linked to variations in the OPA1 gene, which encodes a mitochondrial fusion protein and has been implicated in over 60% of ADOA cases (1, 2). While the primary characteristic of ADOA is its effect on the optic nerve, an increasing number of patients exhibit symptoms extending beyond the ocular domain. This syndromic phenotype is known as ADOA plus (ADOA+) (OMIM #125250) (3, 4), encompassing neurological manifestations like syndromic parkinsonism, cognitive impairments, peripheral neuropathy, and ataxia. Interestingly, about 20% of individuals with an OPA1 variant are classified under ADOA+, highlighting the importance of understanding its pathogenic mechanisms to identify therapeutic solutions.

The pathophysiology of ADOA+ centers on disruptions in mitochondrial dynamics, which are crucial for cellular energy regulation and metabolic adaptation (2). These dynamics are regulated by mitochondrial fusion, which mixes mitochondrial contents, and fission, which separates and eliminates damaged mitochondria via mitophagy (5). Mitophagy, a key component of mitochondrial quality control, is conserved across species (6). Its disruption is linked to neurodegenerative disorders, aging, cancer, and more (7). However, excessive mitophagy can be harmful (8–10).

Variants in OPA1 result in a 50% reduction in gene expression, confirming haploinsufficiency as a key pathogenic mechanism in ADOA. This impairs mitochondrial function and disorganizes mitochondria cristae structures. Kane et al. have linked OPA1 mutations with increased autophagy, whereas mutations causing haploinsufficiency are linked to reduced mitochondrial turnover and autophagy (11). Conversely, Zaninello et al. found higher autophagy levels in retinal ganglion cells expressing mutant OPA1, along with accumulated active AMPK and its autophagic effector ULK1 at the axon hillock (12). Furthermore, Carelli et al. reported that fibroblasts with missense mutations show reduced OPA1 levels and a fragmented mitochondrial network (4). These insights underscore the need for a detailed understanding of how OPA1 variations lead to ADOA+. Thus, therapies targeting OPA1 variants require further research.

Here, we validated an unreported OPA1 variant c.1780T>C using patient-derived fibroblasts with OPA1 haploinsufficiency and a HEK293T cell line with reduced OPA1 expression. Our research shows that OPA1 insufficiency causes significant mitochondrial fragmentation and DRP1 overexpression (OE), disrupting mitochondrial dynamics. This leads to excessive mitophagy, contributing to the ADOA+ phenotype. Interestingly, inhibiting DRP1 with Mdivi-1 offers a potentially new treatment route for ADOA+.

## Results

### Identification of an unreported OPA1 p.F594L gene variant exhibiting haploinsufficiency.

**Figure 1:**
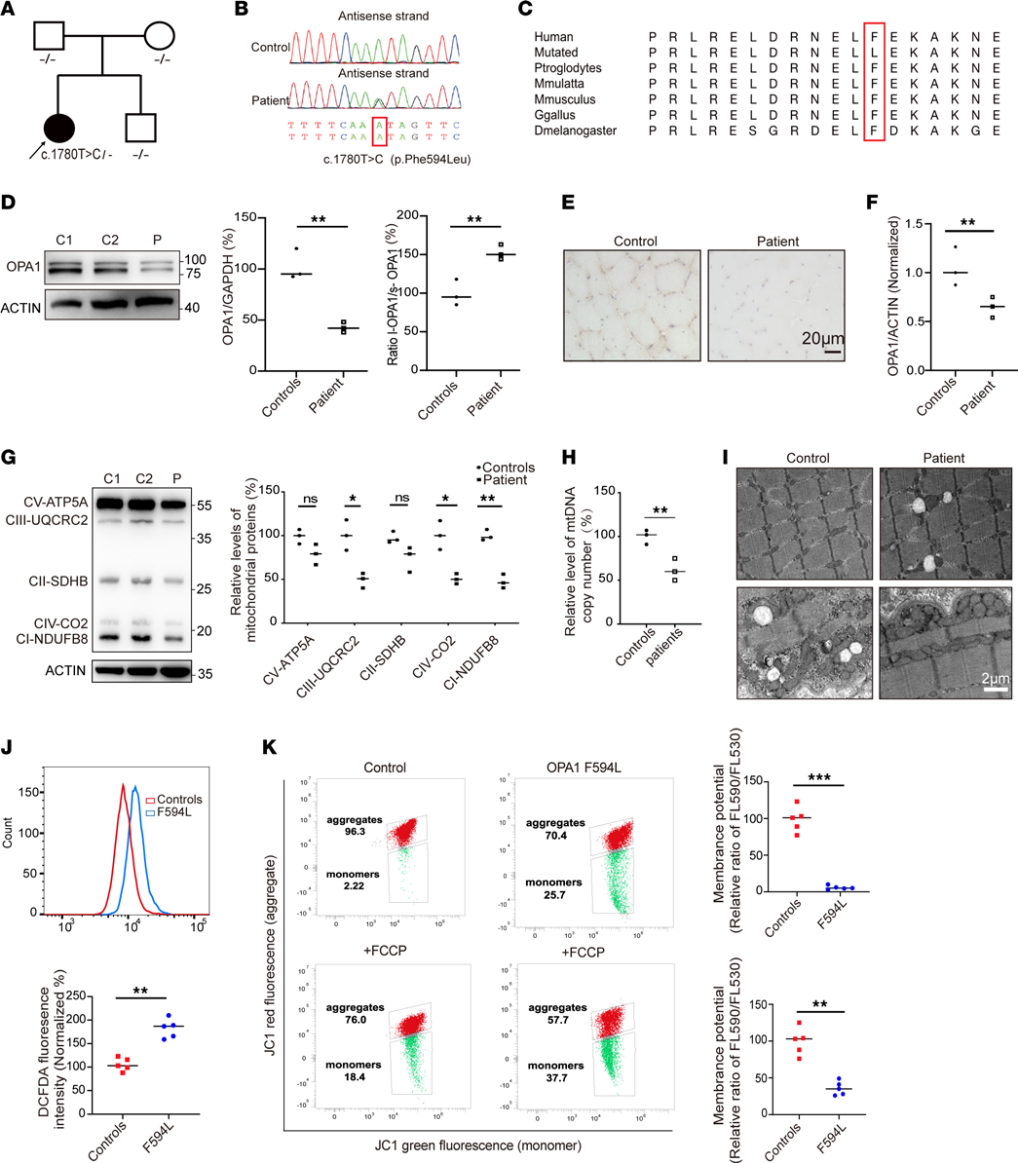
Identification of an unreported p.F594L OPA1 gene variant exhibiting haploinsufficiency. (**A**) The pedigree structure and segregation analysis of variants in families. The arrow indicates the proband. (**B**) DNA sequencing chromatograms comparing the control (upper) and mutant sequences (lower) with the c.1780T>C transition. The variant site is marked with a red box. (**C**) Conservation analysis reveals a high degree of conservation at position Phe594 (boxed in red) in the OPA1 gene across various eukaryotic species. (**D**) Western blot analysis of muscle samples showed markedly lower levels of OPA1 protein in the patient (P) compared with controls (C1, C2), along with an elevated ratio of L-OPA1/S-OPA1 isoforms. Data quantification is shown in the accompanying bar graph. The results are derived from the same samples run on different but concurrent blots. (**E**) IHC analysis showed reduced OPA1 staining in the patient’s muscle samples. Scale bar: 20 μm. (**F**) qPCR analysis revealed downregulation of OPA1 mRNA levels in the patient’s muscle tissue. (**G**) Western blot assays of mitochondrial translation products (ATP5A, UQCRC2, SDHB, NDUFB8, and CO2) in muscle samples. Data quantification indicates mitochondrial complex dysregulation in patient samples. The results are derived from the same samples run on different but concurrent blots. (**H**) The mtDNA copy number analysis of muscle tissues shows a reduction in the patient compared with controls. (**I**) Transmission electron microscopy (TEM) reveals altered mitochondrial morphology with increased subsarcolemmal mitochondrial accumulation in the patient’s muscle tissue compared with controls. Scale bar: 2 μm. (**J**) Flow cytometry analysis using DCFDA indicates elevated ROS levels in patient-derived skin fibroblasts compared with those from controls. (**K**) Mitochondrial membrane potential assessed by JC-1 dye and flow cytometry in control and patient fibroblasts, before and after FCCP treatments. Quantitative analysis indicates reduced membrane potential in the F594L mutant fibroblasts. Statistical analysis was by unpaired, 2-tailed *t* test. *P < 0.05; **P < 0.01; ***P < 0.001 (**D**, **F**, **G**, **H**, **J** and **K**).

Whole-exome sequencing was performed on the proband and their family, resulting in the identification of a c.1780T>C variant in the *OPA1* gene located in the middle domain. This variant was absent in both parents (Figure 1, A and B). No mtDNA deletion was detected in muscle by long-range PCR. A comprehensive global alignment of OPA1 protein sequences from diverse eukaryotic organisms revealed that the p.F594L variant targets a highly conserved residue among vertebrates. The residue, phenylalanine (F) at position 594, is part of a contiguous or nearly invariant group of residues. These domains are conserved across eukaryotes and vertebrates (Figure 1C).

Through visual analysis, it was observed that in the WT OPA1, phenylalanine at position 594 forms hydrogen bonds with asparagine at position 591 and lysine at position 598. Upon mutation to leucine, this residue instead interacts via hydrogen bonds with arginine at position 590 and lysine at position 598. This alteration in hydrogen bonding partners may lead to changes in local protein structure, potentially affecting function (Supplemental Figure 1A; supplemental material available online with this article; https://doi.org/10.1172/jci.insight.180582DS1).

To evaluate the effect of this genetic variation on OPA1 levels, we analyzed protein expression in muscle samples from patients and age- and sex-matched controls using Western blotting. The results revealed a significant reduction in the total levels of OPA1 protein in patient specimens, along with an elevated ratio of long form OPA1 (L-OPA1) to short form OPA1 (S-OPA1) (Figure 1D). Correspondingly, IHC analysis showed a substantial decrease in OPA1 staining in patient samples (Figure 1E). Furthermore, quantitative PCR (qPCR) analysis of muscle samples from patients indicated a decrease in OPA1 mRNA levels (Figure 1F).

### The p.F594L variant in the OPA1 gene caused mitochondrial dysfunction.

OPA1 is vital for the integrity of the respiratory chain, interacting with the respiratory complexes (13). We examined the effect of the OPA1 (p.F594L) variant on the synthesis of respiratory chain complex subunits encoded by nDNA and mtDNA using Western blotting. Patient samples showed a significant decrease in the levels of UQCRC2, NDUFB8, ND4, ND5, CYB, CO2, CO3, and CO4 proteins (Figures 1G and Supplemental Figure 2, B and C). The mtDNA copy number analysis of muscle tissues shows a reduction (Figure 1H). The examination of the patient’s muscle samples through muscle pathology and transmission electron microscopy (TEM) indicated anomalies in mitochondrial morphology, an elevated quantity of subsarcolemmal mitochondria, and muscle fibers deficient in cytochrome c oxidase (COX) (Figure 1I and Supplemental Figure 2A). Mitochondrial function affects intracellular reactive oxygen species (ROS) levels (14). Therefore, we next assessed the effect of the OPA1 variant on ROS levels in skin fibroblasts from patients and controls. DCFDA reagent use indicated a significant increase in ROS in patient cells (Figure 1J). ROS influence oxidative stress and mitochondrial membrane potential (MMP) decline (14). Consequently, MMP levels were evaluated using the JC-1 staining kit, showing a reduction in MMP levels in patients compared with controls; FCCP served as a positive control (Figure 1K).

### OPA1 deficiency decreased the levels of mitochondrial proteins and impaired mitochondrial function.

**Figure 2:**
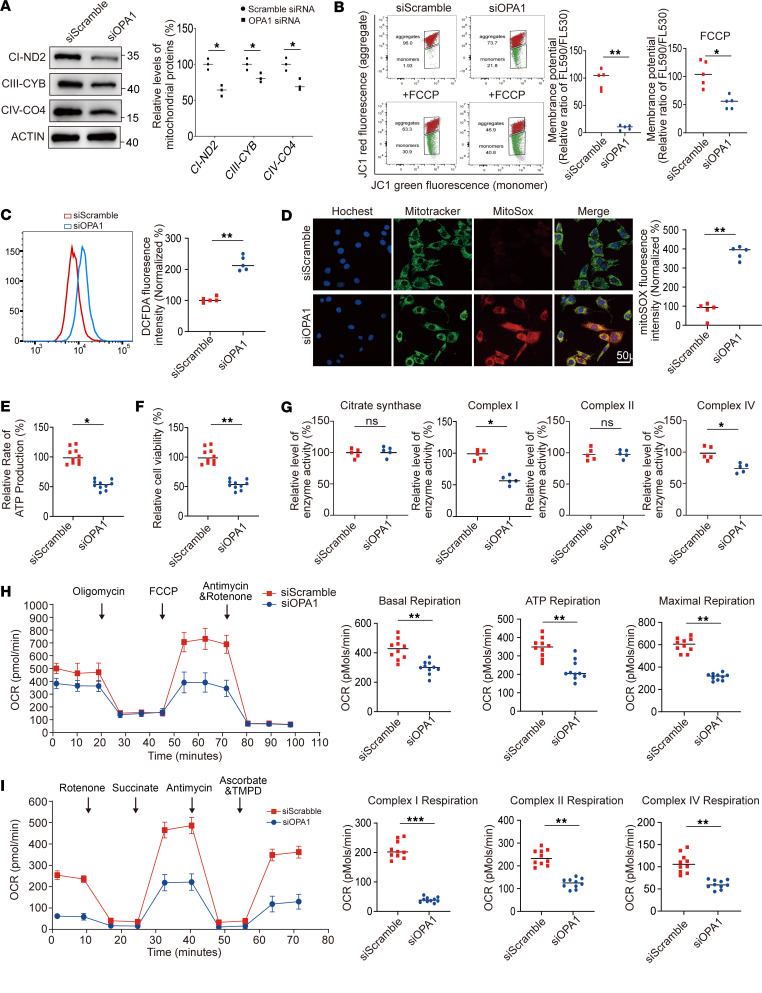
Effect of OPA1 knockdown on mitochondrial proteins, ROS production, and respiratory function. (**A**) Western blot analysis of mitochondrial respiratory complex subunits (ND2, CYB, CO4) in siRNA-mediated OPA1 knockdown (siOPA1) cells compared with scramble siRNA (siScramble) treated cells; decreased protein levels were noted after OPA1 silencing. The results are derived from the same samples run on different but concurrent blots. (**B**) Mitochondrial membrane potential assessed by JC-1 dye in siOPA1 and siScramble cells. Analysis of pre- and post-FCCP treatments demonstrates significantly reduced membrane potential in siOPA1 cells, as indicated by a corresponding decrease in red/green fluorescence ratio. (**C**) Flow cytometric analysis, using DCFDA in siOPA1 and siScramble cells, revealed increased ROS levels in OPA1-deficient cells. (**D**) Fluorescence microscopy images showing mitochondrial network (MitoTracker), mitochondrial ROS production (MitoSOX), and nuclear staining (Hoechst) in siOPA1 and siScramble cells; merged images indicate colocalization. Data quantification in the right panel shows increased MitoSOX intensity in siOPA1 cells. Scale bar: 50 µm. (**E**) ATP assays indicated a significant reduction in ATP levels in siOPA1 cells compared with siScramble cells. (**F**) Cell viability assays indicated decreased viability in siOPA1 cells relative to siScramble cells. (**G**) Quantitative analysis of mitochondrial respiratory complex enzymes (citrate Synthase, complex I, complex II, complex IV) showed decreased Complex I and IV levels in siOPA1 cells. (**H**) Seahorse analysis of oxygen consumption rate (OCR) in siOPA1 and siScramble cells under various metabolic states induced by oligomycin, FCCP, and antimycin A. siOPA1 cells showed reduced basal, ATP-linked, and maximal respiration rates. (**I**) Seahorse OCR measurements following treatment with specific respiratory complex inhibitors (rotenone, antimycin A, and ascorbate, and TMPD) showed reduced OCR, particularly in Complex I–, II–, and IV–driven respiration in siOPA1 cells. Statistical analysis was by unpaired, 2-tailed *t* test. *P < 0.05; **P < 0.01; ***P < 0.001 (**A**–**I**).

To investigate the effect of OPA1 deficiency on mitochondrial function, we introduced OPA1 siRNA and a control plasmid into HEK293T cells. We then performed Western blotting to assess the levels of various subunits of mitochondrial complexes in these cells. The analysis shows that OPA1 deficiency disrupted the functionality of complexes I, III, IV, and V (Figure 2A). Using flow cytometry, we noted a significant reduction of MMP in OPA1 siRNA-transfected HEK293T cells (Figure 2B). Additionally, the DCFDA and MitoSOX probes were used to measure total and mitochondrial ROS levels, respectively. These show elevated ROS levels in OPA1-deficient cells (Figure 2, C and D). The results of the ATP assay indicate a substantial decline in ATP production in OPA1-deficient cells (Figure 2E). The CCK8 assay was used to assess cellular viability, which was decreased in OPA1-deficient cells (Figure 2F). Moreover, we observed a widespread reduction in mitochondrial enzymatic activity (Figure 2G).

Healthy mitochondrial dynamics are essential for mitochondrial respiratory function (5, 15). To investigate the potential effect of OPA1 deficiency on mitochondrial respiratory function, we measured OCR using an extracellular flux analyzer in OPA1 siRNA and control plasmid-transfected HEK293T cells. We found reduced baseline respiration, ATP production, and maximal respiration in cells lacking OPA1 (Figure 2H). We also assessed the respiratory activities facilitated by complexes I, II, and IV using various substrates and inhibitors. This showed a decrease in respiration in OPA1-deficient cells (Figure 2I). These findings link OPA1 deficiency to compromised mitochondrial function, characterized by impaired respiratory complex activity, increased ROS production, and reduced MMP. This deficiency led to proton expulsion from the membrane, reducing ATP production.

### OPA1 deficiency–driven regulation of DRP1 localization and phosphorylation.

**Figure 3:**
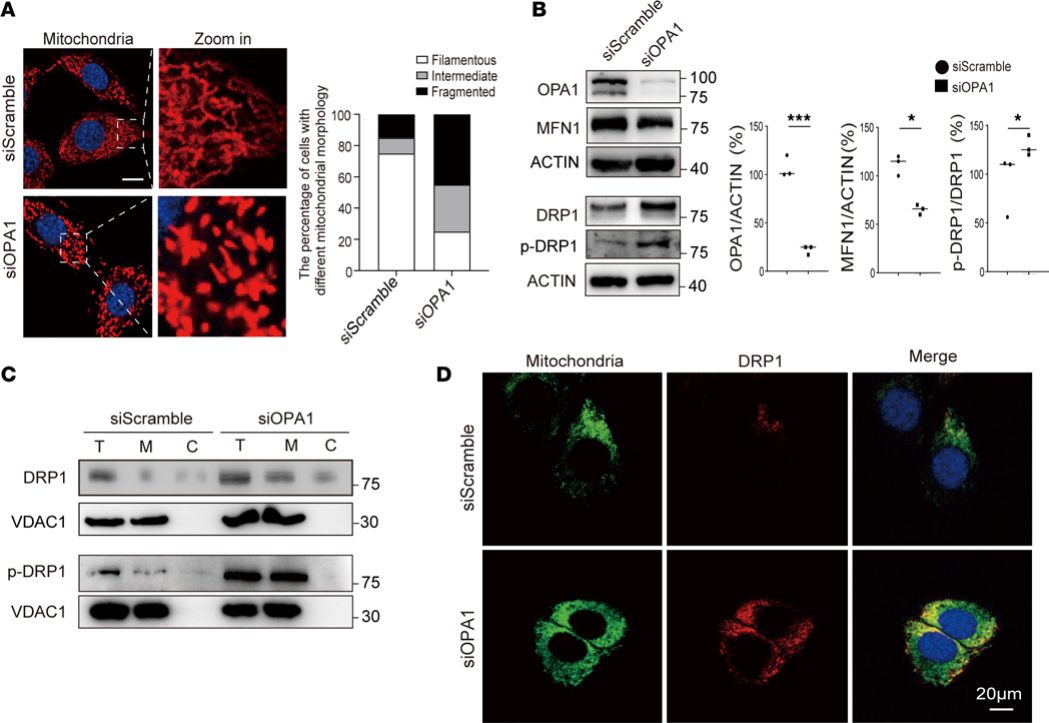
OPA1 knockdown enhanced DRP1 expression and disrupted mitochondrial morphology. (**A**) Confocal microscopy images of mitochondria in MitoTracker stained siScramble and siOPA1 cells. Mitochondrial morphology was filamentous, intermediate, or fragmented mitochondria. Scale bars: 20 μm (inset, 4 μm; **A**) (**B**) Western blot analysis of mitochondrial dynamics proteins OPA1, MFN1, DRP1, and phosphorylated DRP1 (p-DRP1) in siScramble and siOPA1 cells. Data quantification shows a significant decrease in OPA1 and MFN1 levels, with an increase in p-DRP1 levels in siOPA1-treated cells. DRP1 and p-DRP1 results are derived from the same samples run on different but concurrent blots. (**C**) Subcellular fractionation followed by Western blot analysis for DRP1 and p-DRP1, comparing total (T), mitochondrial (M), and cytosolic (C) fractions in siScramble and siOPA1 cells. Results indicate elevated levels of DRP1 and p-DRP1 in the mitochondrial fraction of siOPA1 cells. DRP1 and p-DRP1 run on a separate occasion. (**D**) Confocal microscopy images of siScramble and siOPA1 cells stained for mitochondria and DRP1 show altered DRP1 localization in siOPA1 cells. Scale bar: 20 μm. Statistical analysis was by unpaired, 2-tailed *t* test. *P < 0.05; ***P < 0.001 (**B**).

OPA1 is key for mitochondrial fusion and affects mitochondrial morphology and dynamics (16). To examine mitochondrial morphology, MitoTracker immunofluorescence staining was used. We observed increased mitochondrial fragmentation after OPA1 silencing (Figure 3A). Western blotting showed that OPA1 dysfunction reduces MFN1 expression and increases DRP1 (Figure 3B).

DRP1 is vital for mitochondrial fission and overall dynamics (17). Though studies on OPA1 and DRP1 interaction are limited, some suggest the involvement of FUN14 domain containing 1 (FUNDC1). Our data show that OPA1 absence upregulated DRP1 and mitophagic activity. We isolated mitochondrial and cytosolic components to study changes in DRP1 expression due to OPA1 deficiency. Our results indicate higher mitochondrial DRP1 and its phosphorylated form in OPA1-deficient cells, suggesting DRP1 recruitment to mitochondria and fission activation (Figure 3C). Furthermore, immunofluorescence staining showed DRP1 aggregation at mitochondrial sites in OPA1-deficient cells, unlike the dispersed distribution in control cells (Figure 3D). This highlights the regulatory role of OPA1 in mitochondrial localization and activity of DRP1.

### OPA1 deficiency enhances mitochondrial fission and mitophagy.

**Figure 4:**
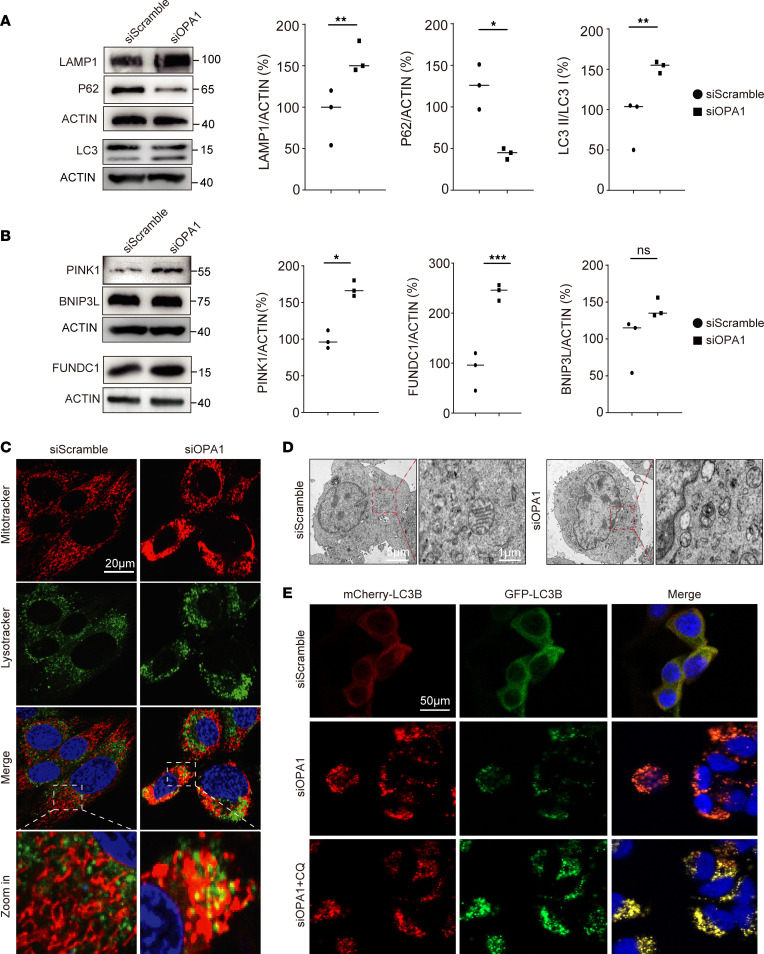
OPA1 participates in molecular interplay with DRP1, and Mdivi-1 mitigates OPA1 deficiency–induced mitophagy dysregulation. (**A**) Western blotting indicates the upregulation of LAMP1 and LC3II and the downregulation of P62 in siOPA1 cells. All genes come from the same samples run on different, but concurrent, blots, except for LC3, which was run on a separate occasion. (**B**) Western blot analysis of mitophagy proteins shows an increase in PINK and FUNDC1 levels in siOPA1 cells compared with siScramble cells. All genes come from the same samples run on different, but concurrent, blots, except for FUNDC1, which was run on a separate occasion. (**C**) MitoTracker and LysoTracker colocalization confocal imaging shows increased mitophagy in siOPA1 cells, indicated by the increased mitochondrial-lysosomal association in magnified panels. (**D**) TEM images of siScramble and siOPA1 cells; the left panels show overall cell morphology, and the right panels provide a magnified view of the mitochondria within the red dashed box. siOPA1-treated cells display disrupted mitochondrial architecture showing increased circular and swollen mitochondria, a hallmark of mitochondrial stress and potential mitophagy. (**E**) Autophagic flux was evaluated using the pCMV-mCherry-GFP-LC3B plasmid, indicating that autophagy is excessively activated by siOPA1 and autophagosome-lysosome fusion is inhibited following treatment with CQ. Statistical analysis was by unpaired, 2-tailed *t* test. *P < 0.05; **P < 0.01; ***P < 0.001 (**A** and **B**). Scale bars: 20 μm (**C**), 5 μm (inset, 1 μm; **D**), 50 μm (**E**).

Mitophagy, a crucial lysosome-dependent degradation process in eukaryotic cells, is vital for mitochondrial homeostasis and quality (18). To study the effect of OPA1 deficiency on autophagy, we analyzed autophagy-related protein levels in OPA1-deficient HEK293T cells by Western blotting. A significant rise in LC3II and LAMP1 levels was noted. In contrast, P62 levels decreased (Figure 4A). Additionally, increased levels of mitophagy-related proteins PINK1 and FUNDC1 were seen. However, BNIP3L levels remained unchanged (Figure 4B). Using MitoTracker and LysoTracker, immunofluorescence staining showed increased mitochondria-lysosome interactions in cells with suppressed OPA1 expression. This indicated higher autophagy activation (Figure 4C). TEM analysis revealed a rise in autolysosomes in cells with lower OPA1 (Figure 4D). Additional research on autophagic flux through plasmid transfection demonstrated that OPA1-deficient cells displayed a higher quantity of red puncta, suggesting an augmented autophagosome generation and maturation in comparison with the control group. The introduction of chloroquine (CQ) effectively suppressed this phenomenon (Figure 4E). These results imply that OPA1 deficiency–induced mitochondrial fusion impairment triggers excessive mitophagy.

### Mdivi-1 counters OPA1 deficiency–induced mitophagy.

**Figure 5:**
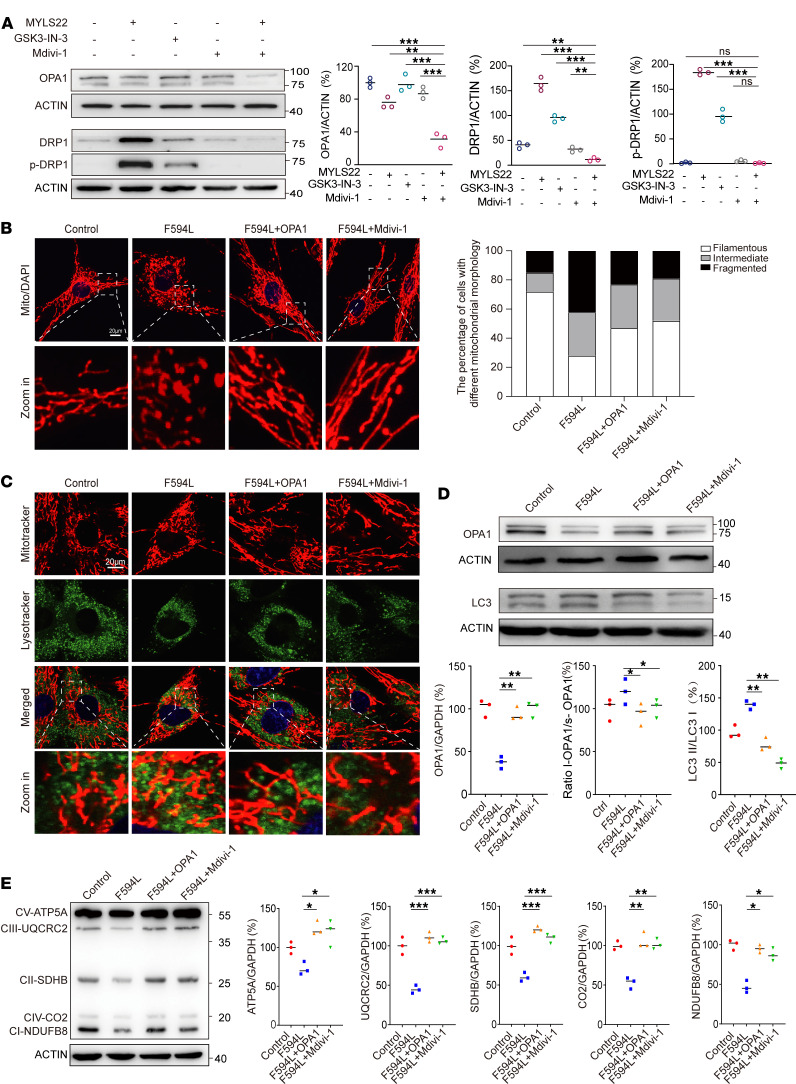
Mdivi-1 ameliorates mitochondrial morphology and autophagy dysregulation and also restores respiratory chain complex protein expression in F594L mutant cells. (**A**) Western blot analysis for OPA1, DRP1, and p-DRP1 levels following treatment with MYLS22, GSK3-IN-3, and Mdivi-1 examining the modulation of these proteins under different pharmacological conditions. Data quantification below the blots shows the relative protein levels normalized to ACTIN. All genes come from the same samples run on different, but concurrent, blots, except for OPA1, which was run on a separate occasion. (**B**) Confocal microscopy illustrated mitochondrial morphology across various conditions: control cells, F594L mutants, F594L mutants with OPA1 lentiviral transduction, and F594L mutants treated with Mdivi-1; all were stained with MitoTracker. The accompanying quantification bar graph shows the proportion of cells exhibiting filamentous, intermediate, or fragmented mitochondria. (**C**) Detailed confocal images showing mitochondrial (red) and lysosomal (green) colocalization. They depict the levels of mitophagy, and zoomed-in views are also provided. (**D**) Western blot analysis was used to quantify the levels of OPA1 and LC3 as well as to assess an increased ratio of L-OPA1/S-OPA1 isoforms, across different treatment groups. The results are derived from the same samples run on different but concurrent blots. (**E**) Western blot analysis of mitochondrial respiratory chain complexes, including CV-ATP5A, CIII-UQCRC2, CII-SDHB, CIV-CO2, and CI-NDUFB8. Data quantification indicated the effects of OPA1 overexpression and Mdivi-1 treatment on the levels of complex expression. The results are derived from the same samples run on different but concurrent blots. Statistical analysis was by 1-way ANOVA and Tukey’s post hoc test. *P < 0.05; **P < 0.01; ***P < 0.001 (**A**, **D**, and **E**). Scale bars: 20 μm (**B**), 20 μm (inset, 4 μm; **C**).

OPA1 deficiency leads to mitochondrial fusion issues, increasing fragmentation and mitophagy. Addressing these issues presents a therapeutic opportunity for related conditions. Mdivi-1, a DRP1 inhibitor, shows potential in mitigating these effects (19, 20). In HEK293T cells, we tested OPA1 inhibitor MYLS22, autophagy agonist GSK3-IN-3, and Mdivi-1. Western blotting revealed DRP1 and p-DRP1 upregulation after MYLS22 use (Figure 5A). Interestingly, MYLS22 and Mdivi-1 coadministration reduced these proteins, indicating the antagonistic effect of Mdivi-1. We then conducted immunofluorescence staining for MitoTracker and LysoTracker in treated cells. Compared with controls, MYLS22 increased colocalization fluorescence, similar to GSK3-IN-3. Hitherto, adding Mdivi-1 with MYLS22 significantly decreased fluorescence. This suggests that Mdivi-1 can effectively suppress MYLS22-induced excessive mitophagy (Supplemental Figure 3).

### Mdivi-1 ameliorates OPA1 deficiency–induced mitochondrial dysfunction.

To investigate the potential therapeutic effects of Mdivi-1 on ADOA+ phenotypes from OPA1 deficiency, we used patient-derived fibroblasts. These cells were subjected to either OPA1 lentiviral OE or Mdivi-1 treatment. We then compared them with untreated control and mutant cells. An initial evaluation of mitochondrial length through fluorescence staining showed that both OPA1 OE and Mdivi-1 treatment caused elongated mitochondrial morphology compared with the mutant group (Figure 5B). Furthermore, immunofluorescence techniques were used to label fibroblasts with MitoTracker and LysoTracker. These techniques indicate a decrease in mitochondria and lysosome colocalization for both treatments (Figure 5C).

Subsequent analysis of OPA1 and LC3 levels via Western blotting revealed increased OPA1 and reduced LC3 levels in cells corrected for OPA1 deficiency or treated with Mdivi-1 (Figure 5D). To assess if inhibiting mitophagy could boost mitochondrial protein expression, we examined levels of nucleus-encoded proteins (ATP5A, UQCRC2, SDHB, NDUFB8, CO4) and mitochondrial-encoded proteins (ND4, CYB, ATP8) by Western blotting. Our study found that correcting OPA1 deficiency and Mdivi-1 administration upregulated mitochondrial subunit proteins relative to the variant group (Figures 5E and Supplemental Figure 4).

**Figure 6:**
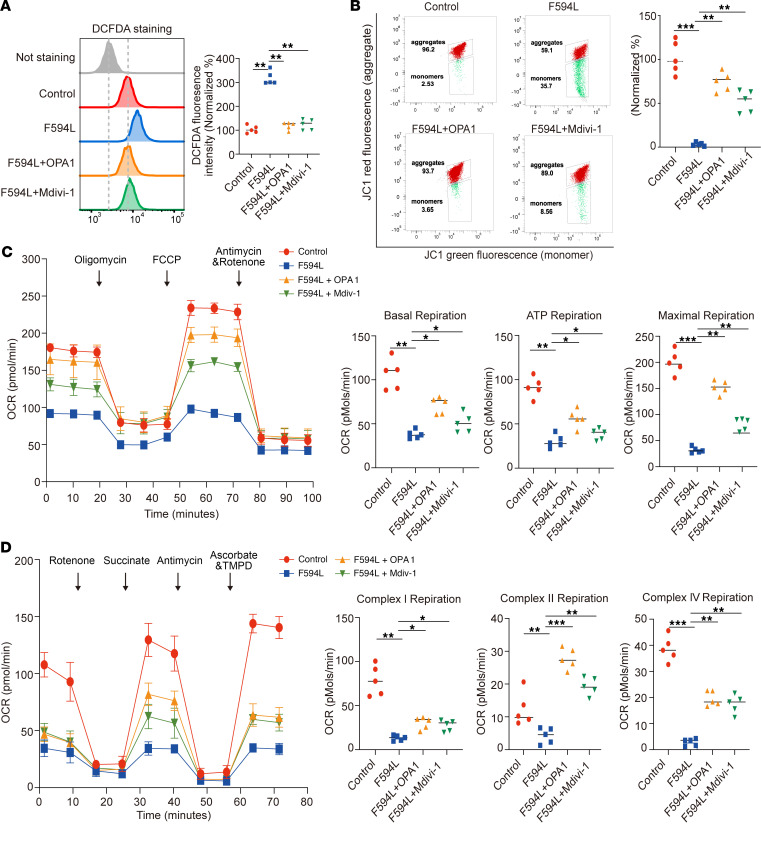
Mdivi-1 attenuates dysfunctions in MMP, ROS accumulation, and OCR in F594L mutant cells. (**A**) Flow cytometry using DCFDA was used to assess the levels of ROS in control, F594L mutant, F594L + OPA1–treated, and F594L + Mdivi-1–treated fibroblasts. The histogram and bar graph demonstrate the relative fluorescence intensity. (**B**) Assessment of MMP using the JC-1 dye across the treatment groups, with flow cytometry plots displaying the proportion of cells with high (aggregates) and low (monomers) MMP. The data quantification bar graph shows the relative intensity of red/green fluorescence. (**C**) Seahorse analysis of OCR in the different treatment groups under basal conditions and in response to oligomycin, FCCP, and antimycin with rotenone. The line graph and accompanying bar graphs detail the OCR during basal respiration, ATP-linked respiration, and maximal respiration. (**D**) Seahorse OCR measurements following treatment with metabolic inhibitors rotenone, succinate, antimycin, ascorbate, and TMPD examined the OCR contributions to individual mitochondrial complexes. Statistical analysis was by 1-way ANOVA and Tukey’s post hoc test. *P < 0.05; **P < 0.01; ***P < 0.001.

To measure the effect on mitochondrial function, we used immunofluorescence and flow cytometry. These methods assessed ROS levels in mitochondria and cells. Both treatments significantly reduced ROS levels compared with the mutant group (Figure 6A and Supplemental Figure 5A). Flow cytometry of JC-1 staining showed improved MMP with OPA1 OE and Mdivi-1 (Figure 6B). ATP assay was used to measure cellular ATP levels, which revealed an increase with both treatments (Supplemental Figure 5B). Using the Seahorse analyzer, we determined elevated basal, ATP-linked, and maximum OCR in treated cells (Figure 6C). Further analysis showed increased OCR of Complex II and IV versus the mutant group (Figure 6D).

## Discussion

In this study, we report the previously unreported c.1780T>C (p. F594L) variant in the OPA1 gene, identified in a 17-year-old female patient presenting with an ADOA+ phenotype. We confirmed its pathogenic nature and highlighted the significant role of OPA1 insufficiency in the excessive activation of mitophagy. Importantly, we demonstrate that both the restoration of OPA1 expression and the use of Mdivi-1 to inhibit heightened mitophagy present potential therapeutic approaches for intervention in ADOA+.

OPA1, a crucial GTPase, is vital for various mitochondrial functions, such as the regulation of mitochondrial dynamics and maintenance of mtDNA (21–23). While ADOA represents the primary clinical manifestation, the spectrum of neurodegenerative disorders encapsulated by ADOA+ has provoked substantial scientific inquiry (24). Since the initial report in 2003 (25), there has been a growing focus on understanding the variant spectrum of ADOA+.

To date, the ClinVar database has registered 591 OPA1 variants, presenting a wide range of clinical manifestations. These variants are classified into several types: missense (206, 24%), canonical-splice (117, 13%), frameshift (127, 15%), deletions/insertions (69, 8%), and nonsense (72, 14%). Among these, 61 variants have been specifically linked to ADOA+, with 58 variants explicitly linked to the ADOA+ phenotype. These variants are distributed throughout the OPA1 gene, with a notable concentration in the GTPase (19, 32.8%) and the middle/dynamin domains (21, 36.2%), suggesting these regions as potential hotspots for variants. In 2023, Cartes-Saavedra et al. (26) found that OPA1 disease-causing mutants exhibit domain-specific effects on mitochondrial ultrastructure and fusion. The GTPase effector domain (GED) is not necessary for fusion or OPA1 oligomer formation, but it is crucial for GTPase activity. The middle domain plays a role in regulating OPA1 self-assembly and may be closely linked to OPA1 function (13, 26). Therefore, a comprehensive understanding of the mechanisms underlying various OPA1 mutations and their genotype-phenotype correlations is essential for elucidating the pathogenesis of ADOA+.

The c.1780T>C (p. F594L) variant has alteration at the 594 position in the amino acid sequence, specifically from phenylalanine to leucine. Patient-derived muscle samples and fibroblasts expressing this variant exhibit disrupted mitochondrial morphology and compromised translation and respiratory functions. This disruption reduces the mitochondrial proton electrochemical gradient, disrupts MMP, and elevates ROS levels, thereby causing excessive mitophagy. The upregulated mitophagy, together with OXPHOS defects, causes detrimental oxidative stress within the mitochondria. These sequential events ultimately contribute to the development of ADOA+.

Mitochondrial dynamics, involving the coordinated interplay of fusion and fission, exert a substantial influence on the morphology, size, and quantity of mitochondria (27, 28). The process of mitochondrial fusion benefits the maintenance of mitochondria by facilitating the exchange of matrix and membrane components among partially damaged mitochondria. This exchange serves as a mechanism for rescuing and replenishing mitochondria (29). Conversely, an overabundance of fission or a reduction in mitochondrial fusion can result in the fragmentation of mitochondria. This imbalance triggers excessive activation of mitophagy, hindering the fusion-dependent restoration of impaired mitochondria. Consequently, cells are compelled to undergo an excessive process of self-digestion, thereby aggravating mitochondrial dysfunction (8, 16).

Investigations into the pathophysiology of mammals have revealed that an upsurge in autophagy may lead to neuronal damage under conditions such as neonatal, immune dysfunction, and myocardial cell ischemia (30–32). Moreover, the suppression of autophagy has been identified as a promising strategy in the realm of cancer therapy (10, 33). In the context of ADOA, Zaninello et al., using mice with an RGC-specific OPA1 deficiency, discovered that eliminating the autophagy-related gene *Atg7* mitigates the heightened autophagy and ameliorates the vision defects induced OPA1 deficiency (12). The action mechanism of phenanthroline appears to mirror the mitochondrial malfunctions triggered by a lack of OPA1. This metalloprotease inhibitor impedes the processing of OPA1, initiating an overactive mitophagy and causing a significant decrease in mitochondria and mtDNA (34). Notably, our investigation highlights the surge in DRP1 expression alongside OPA1 deficiency. Typically, a balanced interaction between OPA1 and DRP1 is crucial for sustaining mitochondrial balance. The reduction of OPA1 seems to upset this balance, affecting DRP1 expression by altering their mutual interaction. Chen et al. (35) demonstrated the interaction between OPA1 and DRP1 with FUNDC1, revealing that mitochondrial distress reduces the connection between OPA1 and FUNDC1 but strengthens it with DRP1 (36). Schuettpelz et al. have shown that SLC25A46 colocalizes with OPA1 and DRP1, crucially influencing mitochondrial division and amalgamation (37). These insights shed light on the complex dynamics between OPA1 and DRP1. Mdivi-1, derived from quinazolinone, is now a recognized inhibitor of DRP1. Recent explorations into its therapeutic potential across various diseases have been enlightening. For instance, Deng et al. showed how Mdivi-1 counters angiotensin II–induced hypertension by facilitating the transformation of vascular smooth muscle cells (38). Aishwarya et al. uncovered that Mdivi-1 blocks cardiomyocyte macroautophagy and enzymatically splits L-OPA1 (19). Additionally, Wang’s group, through experiments on mouse models of Alzheimer’s disease, illustrated that Mdivi-1 notably diminishes mitochondrial fragmentation and boosts energy balance, thereby enhancing cognitive functions related to learning and memory. Importantly, Mdivi-1 has also been observed to obstruct the buildup of amyloid-β (Aβ) plaques within the brain (39). Concurrently, research from the Carreras group underscores Mdivi-1’s efficacy in remedying fat tissue issues tied to obesity and diabetes by blocking mitochondrial division triggered by Drp1 (40).

Beyond its diverse therapeutic effects, Mdivi-1 has shown potential in slowing autoimmune encephalomyelitis progression. It achieves this by reducing inflammatory cell invasion in the spinal cord and combating demyelination caused by inflammation (41). Aishwarya et al. pointed out that inhibitors of Drp1, like Mdivi-1, could bolster cardiac function and diminish symptom severity in heart diseases (19). Bordt et al. reported that Mdivi-1 notably boosts the expression of crucial mitochondrial merging proteins, such as Mfn1, Mfn2, and OPA1 (42). Yang et al. identified a compound that targets DRP1, effectively preventing mitochondrial fragmentation induced by toxins. It also reinstated normal mitochondrial shape in cells with pathogenic OPA1 variants showing fusion deficits (36). Overall, these findings suggest the potential effects of Mdivi-1 on both mitochondrial fission and fusion.

Using Mdivi-1 intervention, we found that Mdivi-1 treatment increased OPA1 expression in OPA1-variant cells. Concurrently, this led to a pronounced inhibition of excessive mitophagy, ameliorating the OPA1 variant–associated cellular functional impairments. Notably, we could successfully correct the genetic anomaly in OPA1-mutant cells using lentiviral OE. This indicated that the therapeutic benefits of Mdivi-1 treatment closely resembled those of genetic intervention. Our study reveals a promising and innovative therapeutic approach for ADOA+.

## Methods

### Sex as a biological variable.

Our study involved only 1 patient, and sex was not considered as a biological variable.

### Patients.

A 17-year-old female proband underwent assessment for bilateral visual impairment and bilateral hand tremors. The examination revealed the presence of a high-arched palate, reduced arm swing, a dragging gait, ataxia as demonstrated by the finger-to-nose test, and bradykinesia during rapid alternating movements. Endocrine evaluations indicated elevated levels of follicle-stimulating hormone (FSH) and luteinizing hormone (LH). Standard hematology, urinalysis, and metabolic tests yielded no significant findings. Electromyography findings suggested the presence of peripheral neuropathy in the lower limbs. Cognitive function was found to be below the normative range, with scores of 19 on the MMSE and 14 on the MoCA. Ultrasonography of the pelvis indicated atrophic uterus and ovaries. Given the patient’s presentation of various systemic symptoms, such as parkinsonian syndrome, endocrine abnormalities, and cognitive impairment, in addition to optic neuropathy, we consider that this individual exhibit an ADOA+ phenotype.

### Genetic analysis and variant detection.

Genomic DNA was extracted from peripheral blood samples utilizing the DNA Isolation Kit (Blood DNA Kit V2, CW2553). Subsequently, DNA libraries were subjected to sequencing on the illumina novaseq platform, generating paired-end 200 bp reads. The obtained data were aligned to the human reference genome (GRCh19/hg19) and subjected to variant analysis using BWA-MEM (Burrows-Wheeler Aligner - Maximal Exact Matches) and Sentieon, a software package for optimized DNA sequence analysis. To validate the identified candidate mutations, Sanger sequencing was employed.

### Protein structural analysis.

The predicted structures for both the WT and mutant forms of OPA1 were acquired from the AlphaFold Protein Structure Database (https://alphafold.ebi.ac.uk/). A comparative structural analysis was conducted using the molecular visualization software PyMOL to identify conformational differences.

### Cell culture and reagent treatments.

Skin fibroblasts obtained from patients, along with HEK293T cells sourced from the American Type Culture Collection (ATCC), constituted the primary cell types utilized in our research endeavors. The aforementioned cell cultures were sustained in DMEM (Thermo Fisher Scientific), supplemented with 10% v/v FBS (Thermo Fisher Scientific), and penicillin/streptomycin (100 U/mL; MilliporeSigma). The cells were maintained under controlled environmental conditions, encompassing a temperature of 37°C, 5% CO_2_, and a humidified atmosphere. A variety of pharmacological interventions were utilized in cellular treatment interventions, employing different concentrations and durations. These interventions included the administration of FCCP at a concentration of 10 μM for a duration of 30 minutes to induce oxidative stress, the application of MYLS22 at a concentration of 50 μM for a duration of 2 hours to inhibit OPA1 expression, the use of GSK3-IN-3 at a concentration of 2.5 μM for a duration of 16 hours to enhance autophagy, and the treatment with Mdivi-1 at a concentration of 5 μM for a duration of 16 hours to suppress mitophagy.

### siRNA transfection and lentiviral transduction.

HEK293T and skin fibroblast cells were cultured in 6-well plates or 8-well ibidi μ-slides until they reached a confluence of approximately 60%–70%. The transfection procedure utilized Lipofectamine 2000 (Invitrogen, L11668019) in accordance with the manufacturer’s provided guidelines. Either OPA1 siRNA (sense, 5′-GGUGAGAAGAAGAUUAAAUTT-3′, and antisense, 5′-TCATGCTCTTTCCCTTTCGGT-3′) or scramble siRNA were introduced into the cells based on the specific requirements of the experiment. Following the transfection process, the cells were incubated in a controlled environment at 37°C and 5% CO_2_ for 48 hours to enhance gene silencing. OPA1 OE was achieved in skin fibroblasts through lentiviral transduction. A recombinant lentivirus carrying the OPA1 gene was utilized for this purpose. After exposure to the viral medium, the cells were incubated at 37°C in a 5% CO_2_ atmosphere for 24 hours, followed by replacement with fresh culture medium for 48 hours to facilitate OPA1 OE.

### Histopathological analysis.

Muscle tissues were cryosectioned according to established protocols to generate 8 μm–thick sections tailored for specific histological assays. Subsequently, these sections were stained utilizing COX staining and a modified Gomori trichrome (MGT) technique. IHC analysis was conducted using a specific OPA1 antibody (Proteintech, 27733-1-AP, 1:100), with signal detection facilitated by the HRP/DAB (ABC) IHC Kit (Abcam, ab64264, 1:1,000) compatible with primary antibodies from both mouse and rabbit sources.

### qPCR analysis.

The total RNA extraction from cell samples was conducted using the RNA Extraction Kit (Vazyme, RM201-02) following the manufacturer’s instructions. Subsequently, 500 ng of the RNA was employed for cDNA synthesis using the Hiscript I Q Select RT SuperMix Kit (Vazyme, R233-01). For gene expression quantification, real-time PCR was performed using SYBR qPCR Master Mix (Vazyme, Q712-02/03). The primer sequences used were as follows: OPA1, sense, 5′-AATAACTATCCTCGCCTGCGGT-3′, and antisense, 5′-TCATGCTCTTTCCCTTTCGGT-3′; GAPDH, sense, 5′-CAGGTTGTCTCCTGCGACTTC-3′, and antisense, 5′-GGGTGGTCCAGGGTTTCTTAC-3′; COX, sense, 5′-CAGCCCATGACCCCTAACAG-3′, and antisense, 5′-TACATCGCGCCATCATTGGT-3′.

### TEM.

Cellular ultrastructure was analyzed using TEM. Initially, cells were immobilized with a mixture of 3% glutaraldehyde and 2% paraformaldehyde in 0.1M cacodylate buffer (pH 7.4) for a duration of 2 hours at room temperature. Subsequently, a secondary fixation was performed using 1% osmium tetroxide in the same buffer for 1 hour. After fixation, a series of dehydration steps was carried out on the cells, followed by their embedding in a suitable resin. Ultrathin sections measuring 60–80 nm were then obtained using an ultramicrotome and placed on copper grids. The prepared sections were examined under a TEM (Hitachi H-7800) at an appropriate magnification to visualize the cellular projections.

### Mitochondrial and cytosolic fractionation.

Mitochondrial and cytosolic fractions were obtained from HEK293T cells using the Mitochondria Isolation Kit (Beyotime, C3601). The cells were homogenized with a glass Potter-Elvehjem homogenizer equipped with a Teflon pestle, resulting in a homogenate. This homogenate was initially centrifuged at 800*g* for 10 minutes at 4°C to separate cell debris. The supernatant was then subjected to a second centrifugation at 10,000*g* for 15 minutes at 4°C. The pellet obtained, consisting mainly of the mitochondrial fraction, was subjected to 2 rinses using the designated isolation buffer. The resulting supernatant, containing an enriched cytosolic fraction, was meticulously collected.

### Mitochondrial enzyme activity.

Mitochondrial enzyme activities were evaluated using the Abbkine Mitochondrial Enzyme Activity Assay Kit (KTB1850, KTB1860, KTB1880). The protocol encompassed multiple sequential steps to ensure precise measurement of enzymatic activities. Initially, isolated mitochondria were subjected to incubation in a designated assay buffer to enhance mitochondrial enzyme activity. Subsequently, the specific substrate for the enzyme of interest was introduced, and the resulting mixture was incubated at 37°C for a predetermined duration, as per the manufacturer’s guidelines. The observed colorimetric alteration in the reaction mixture served as an indicator of enzyme activity.

### Western blot analysis.

Cellular and tissue samples were lysed using RIPA assay lysis buffer (Beyotime), supplemented with 1% protease and 1% phosphatase inhibitors. The resulting lysates were subjected to immunoblotting, following a protocol described in previous studies conducted by our research group. The primary antibodies utilized in the experiment included OXPHOS cocktail (Abcam, ab110411, 1:1,000), ND2 (Proteintech, 19704-1-AP, 1:1,000), ND4 (Abclonal, A9941, 1:1,000), ND5 (Proteintech, 55410-1-AP, 1:1,000), VDAC1 (Abcam, ab15895, 1:1,000), CYB (Proteintech, 55090-1-AP, 1:1,000), CO3 (Proteintech, 55082-1-AP, 1:1,000), CO4 (Proteintech, 11242-1-AP, 1:1,000), ATP6 (Proteintech, 55313-1-A, 1:1,000), ATP8 (Proteintech, 26123-1-AP, 1:1,000), OPA1 (Proteintech, 27733-1-AP, 1:1,000), MFN1 (Proteintech, 13798-1AP, 1:1,000), DRP1 (Proteintech, 12957-1-AP, 1:1,000), p-DRP1 (Abclonal, AP1353, 1:1,000), P62 (Abcam, ab91526, 1:1,000), LC3 (Proteintech, 14600-1-AP, 1:1,000), LAMP1 (Proteintech, 21997-1-AP, 1:1,000), PINK1 (Proteintech, 23247-1-AP, 1:1,000) BNIP3L (Proteintech, 12986-1-AP, 1:1,000), FUNDC1 (Abclonal, A22001, 1:1,000), β-actin (Abcam, ab8226, 1:3,000), and GAPDH (Abcam, ab8245, 1:3,000). After incubating the samples with primary antibodies, they were subsequently treated with secondary antibodies conjugated to HRP that were specific for either rabbit or mouse immunoglobulins. The visualization of protein bands was achieved by employing Western ECL Substrate (MilliporeSigma), and the resulting images were captured using a Tanon 5500 camera system.

### Cell viability assessment.

Cell viability was assessed using the Cell Counting Kit-8 assay (CCK-8; Beyotime, C0038) following the manufacturer’s instructions. HEK293T cells were seeded in 96-well plates at a density of 5,000 cells per well and allowed to adhere overnight. Subsequently, the CCK-8 reagent was added to each well and incubated for 2 hours at 37°C. Absorbance values were measured at 450 nm using a microplate reader (BioTek). The resulting cell viability was expressed as a percentage relative to the control cells.

### Immunofluorescence staining.

Cells were plated onto glass coverslips at a density of 2 × 10^4^ cells per well and allowed to adhere overnight. Subsequently, the cells were permeabilized using a 0.1% Triton X-100 solution for a duration of 5 minutes, followed by a blocking step with 5% BSA for an additional 20 minutes. This was followed by the application of primary antibodies specific to DRP1 (Proteintech, 12957-1-AP, 1:100) and mitochondria (Abcam, ab92825, 1:500). To stain the nuclei, DAPI (Abcam, ab104139, 1:100) was also applied. Subsequent to the primary staining, appropriate secondary antibodies were administered. After the antibody incubations and subsequent washing procedures, the coverslips were meticulously mounted onto slides utilizing a DAPI-containing antifade mounting medium. Confocal microscopy (Leica) was utilized to capture high-resolution images of the cells.

### Mitochondrial morphology.

The morphology of mitochondria was examined in HEK293T and skin fibroblast cells. The cells were cultured to achieve a confluency of 40%–50% in 8-well ibidi μ-slides. After a 24-hour incubation period, MitoTracker Red staining (Invitrogen, M22426) was applied to the cells at a final concentration of 100 nM in a reduced medium. The cells were then incubated at 37°C for 20 minutes. Subsequently, the cells were washed twice to remove any residual, nonincorporated dye, ensuring accurate visualization of only stained mitochondria. Images of the cells were captured using confocal microscopy. The software’s fiber length feature was utilized to measure the length of the mitochondria directly from the acquired images.

### Autophagy flux assessment.

Autophagy flux analysis was conducted in HEK293T cells utilizing the pCMV-mCherry-GFP-LC3B plasmid (Beyotime, D2816). Enhanced autophagy was indicated by the presence of both yellow (representing colocalization of GFP and mCherry fluorescence) and red puncta. HEK293T cells cultured on coverslips were transfected with the plasmid using Lipofectamine 2000 following the manufacturer’s instructions. The cells were combined treated with either scramble siRNA, OPA1 siRNA, or a combination of OPA1 siRNA and CQ (50 μM, 12 hours).

### Measurement of ATP levels.

ATP synthesis was evaluated by employing the ATP Assay Kit (Beyotime, S0027). In total, 1 × 10^6^ cells were lysed in 200 μL of ATP assay buffer, and subsequently, 10 μL of the resulting lysate was combined with 90 μL of the ATP reaction mixture in a 96-well plate. The luminescence was subsequently quantified in accordance with the manufacturer’s guidelines. A standard curve was constructed using ATP standards provided within the kit, which facilitated the determination of ATP concentrations in the experimental samples. The final outcomes were expressed in terms of ATP concentration.

### Evaluation of mitochondrial bioenergetics.

The Agilent Seahorse XFe24 Analyzer was employed to investigate mitochondrial bioenergetics. HEK293T cells at a density of 4 × 10^4^, and skin fibroblast cells at a density of 1.5 × 10^4^ were cultured in Seahorse XFe24 plates and subjected to both a Mito Stress Test and a Respiratory Complex Test. The Mito Stress Test involved the sequential administration of 1 μM oligomycin, 1 μM FCCP, 0.5 μM rotenone, and 0.5 μM antimycin, enabling the determination of basal, maximal, and ATP-coupled oxygen consumption rate (OCR) values. In the Respiratory Complex Test, a series of respiratory chain inhibitors were subsequently introduced, including 2 μM rotenone, 10 mM succinate, 5 μM antimycin A, 10 mM ascorbate, and 0.5 mM TMPD.

### Measurement of ROS level.

The assessment of ROS production involved the utilization of DCFH-DA (Solarbio, CA1410) and MitoSOX Red reagent (Invitrogen, M36008). To measure mitochondrial ROS, HEK293T and skin fibroblast cells were cultured on 8-well ibidi μ-slides until reaching appropriate confluence. Once adhered, cells were incubated with MitoSOX Red reagent at a concentration of 5 μM for a duration of 15 minutes, maintaining standard culture conditions (37°C, 5% CO_2_). Following staining, cells were thoroughly washed, and confocal microscopy was employed to capture the fluorescence. To quantify the overall intracellular ROS levels, cellular samples were obtained and resuspended in phosphate-buffered saline (PBS) at a concentration of 1 × 10^6^ cells/mL. Subsequently, the cells were subjected to staining with a 5 μM concentration of DCFH-DA reagent for a duration of 15 minutes. Following the incubation period, the cells were thoroughly washed to eliminate any residual unbound dye. Subsequent analysis of fluorescence intensity was performed using flow cytometry.

### MMP detection.

The MMP was measured using the JC-1 Mitochondrial Membrane Potential Assay Kit (Beyotime, C2006). HEK293T and skin fibroblast cells were collected and resuspended in culture medium to achieve a density of 1 × 10^6^ cells/mL. The cells were then treated with JC-1 dye (10 μg/mL) and incubated at 37°C for 20 minutes in a 5% CO_2_ incubator. After incubation, the cells were carefully washed twice with staining buffer to remove excess dye. The fluorescence signals of JC-1 monomers (excitation/emission: 530/488 nm) and aggregates (excitation/emission: 575/590 nm) were recorded. FCCP (10 μM), a potent disruptor of mitochondrial oxidative phosphorylation, was employed as a positive control.

### Statistics.

Difference among groups were analyzed by 2-tailed unpaired Student’s *t* test and with a significance threshold of *P <* 0.05. All assays were repeated at least 3 times. Statistical analyses were performed using either Prism software version 9.0.

### Study approval.

All procedures involving the patient were approved by the Medical Ethics Committee of Qilu Hospital. Written informed consent was obtained from the patient prior to participation.

### Data availability.

Values for all data points in graphs are reported in the Supporting Data Values file.

## Author contributions

YL designed and performed most of the experiments; DW, BL, JW, LX, and XS helped with experiments; KJ, CY, FL, and YZ supervised the study; YL wrote the paper, with critical edits from CY, FL, and YZ. The authors read and approved the final manuscript.

## Supplementary Material

Supplemental data

Unedited blot and gel images

Supporting data values

## Version 1 06/25/2024

In-Press Preview

## Version 2 08/08/2024

Electronic publication

## References

[1] Yu-Wai-Man P, Chinnery PF (2013). Dominant optic atrophy: novel OPA1 mutations and revised prevalence estimates. Ophthalmology.

[2] Lenaers G (2021). Dominant optic atrophy: Culprit mitochondria in the optic nerve. Prog Retin Eye Res.

[3] Baker MR (2011). Subclinical multisystem neurologic disease in “pure” OPA1 autosomal dominant optic atrophy. Neurology.

[4] Carelli V (2015). Syndromic parkinsonism and dementia associated with OPA1 missense mutations. Ann Neurol.

[5] Archer SL (2013). Mitochondrial dynamics--mitochondrial fission and fusion in human diseases. N Engl J Med.

[6] Choi AM (2013). Autophagy in human health and disease. N Engl J Med.

[7] Lou G (2020). Mitophagy and neuroprotection. Trends Mol Med.

[8] Cao Y (2023). A mitochondrial SCF-FBXL4 ubiquitin E3 ligase complex degrades BNIP3 and NIX to restrain mitophagy and prevent mitochondrial disease. EMBO J.

[9] Zeng X (2022). Activated Drp1 regulates p62-mediated autophagic flux and aggravates inflammation in cerebral ischemia-reperfusion via the ROS-RIP1/RIP3-exosome axis. Mil Med Res.

[10] Zhou J (2015). A novel autophagy/mitophagy inhibitor liensinine sensitizes breast cancer cells to chemotherapy through DNM1L-mediated mitochondrial fission. Autophagy.

[11] Kane M (2017). Autophagy controls the pathogenicity of OPA1 mutations in dominant optic atrophy. J Cell Mol Med.

[12] Zaninello M (2020). Inhibition of autophagy curtails visual loss in a model of autosomal dominant optic atrophy. Nat Commun.

[13] von der Malsburg A (2023). Structural mechanism of mitochondrial membrane remodelling by human OPA1. Nature.

[14] Shadel GS, Horvath TL (2015). Mitochondrial ROS signaling in organismal homeostasis. Cell.

[15] Giacomello M (2020). The cell biology of mitochondrial membrane dynamics. Nat Rev Mol Cell Biol.

[16] Chan D (2020). Mitochondrial dynamics and its involvement in disease. Annu Rev Pathol.

[17] Rosdah AA (2020). New perspectives on the role of Drp1 isoforms in regulating mitochondrial pathophysiology. Pharmacol Ther.

[18] Wang S (2023). The mitophagy pathway and its implications in human diseases. Signal Transduct Target Ther.

[19] Aishwarya R (2020). Pleiotropic effects of mdivi-1 in altering mitochondrial dynamics, respiration, and autophagy in cardiomyocytes. Redox Biol.

[20] Li YH (2019). Mdivi-1, a mitochondrial fission inhibitor, modulates T helper cells and suppresses the development of experimental autoimmune encephalomyelitis. J Neuroinflammation.

[21] Quintana-Cabrera R (2021). Opa1 relies on cristae preservation and ATP synthase to curtail reactive oxygen species accumulation in mitochondria. Redox Biol.

[22] Lai Y (2020). Restoration of L-OPA1 alleviates acute ischemic stroke injury in rats via inhibiting neuronal apoptosis and preserving mitochondrial function. Redox Biol.

[24] Ham M (2019). Meta-analysis of genotype-phenotype analysis of OPA1 mutations in autosomal dominant optic atrophy. Mitochondrion.

[25] Alward WLM (2003). The OPA1 gene and optic neuropathy. Br J Ophthalmol.

[26] Cartes-Saavedra B (2023). *OPA1* disease-causing mutants have domain-specific effects on mitochondrial ultrastructure and fusion. Proc Natl Acad Sci U S A.

[27] Quintana-Cabrera R, Scorrano L (2023). Determinants and outcomes of mitochondrial dynamics. Mol Cell.

[28] Ni H (2015). Mitochondrial dynamics and mitochondrial quality control. Redox Biol.

[29] Youle R, van der Bliek A (2012). Mitochondrial fission, fusion, and stress. Science.

[30] Xue H (2021). Dexmedetomidine post-conditioning ameliorates long-term neurological outcomes after neonatal hypoxic ischemia: The role of autophagy. Life Sci.

[31] Ajoolabady A (2021). Targeting autophagy in ischemic stroke: From molecular mechanisms to clinical therapeutics. Pharmacol Ther.

[32] Patoli D (2020). Inhibition of mitophagy drives macrophage activation and antibacterial defense during sepsis. J Clin Invest.

[33] Li J (2021). Regulation and function of autophagy in pancreatic cancer. Autophagy.

[34] Diot A (2015). A novel quantitative assay of mitophagy: Combining high content fluorescence microscopy and mitochondrial DNA load to quantify mitophagy and identify novel pharmacological tools against pathogenic heteroplasmic mtDNA. Pharmacol Res.

[35] Chen M (2016). Mitophagy receptor FUNDC1 regulates mitochondrial dynamics and mitophagy. Autophagy.

[36] Yang J (2023). Chemical inhibition of mitochondrial fission via targeting the DRP1-receptor interaction. Cell Chem Biol.

[37] Schuettpelz J (2023). The role of the mitochondrial outer membrane protein SLC25A46 in mitochondrial fission and fusion. Life Sci Alliance.

[38] Deng Y (2021). Mdivi-1, a mitochondrial fission inhibitor, reduces angiotensin-II- induced hypertension by mediating VSMC phenotypic switch. Biomed Pharmacother.

[39] Wang W (2017). Inhibition of mitochondrial fragmentation protects against Alzheimer’s disease in rodent model. Hum Mol Genet.

[40] Finocchietto P (2022). Inhibition of mitochondrial fission by Drp-1 blockade by short-term leptin and Mdivi-1 treatment improves white adipose tissue abnormalities in obesity and diabetes. Pharmacol Res.

[41] Li Y (2019). Mdivi-1, a mitochondrial fission inhibitor, modulates T helper cells and suppresses the development of experimental autoimmune encephalomyelitis. J Neuroinflammation.

[42] Bordt E (2017). The Putative Drp1 Inhibitor mdivi-1 is a reversible mitochondrial complex I inhibitor that modulates reactive oxygen species. Dev Cell.

