# Supplementary material for: Targeting DRP1 with Mdivi-1 to correct mitochondrial abnormalities in ADOA+ syndrome
Source: JCI Insight. 2024 Jun 25;9(15):e180582. doi: 10.1172/jci.insight.180582 (PMC11383607; doi:10.1172/jci.insight.180582)
Supplement: Supplemental data [file jciinsight-9-180582-s134.pdf]

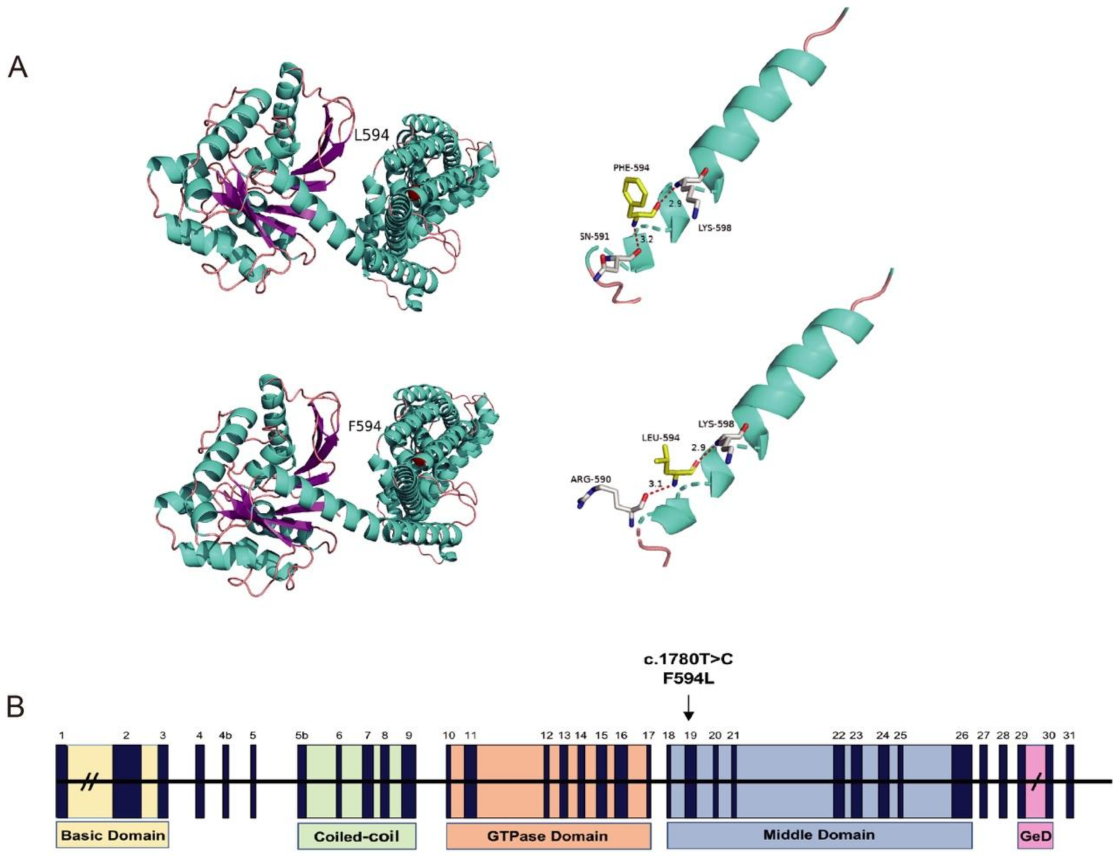

Supplementary Figure 1. Predicted structural alterations and domain architecture of the OPA1 protein. (A) Structural models derived from AlphaFold predicting the change in conformations of wild-type and mutant OPA1 proteins. The wild-type structure (top) has hydrogen bonding of phenylalanine 594 position with nearby residues. This is altered in the mutant structure (bottom)**. (B)** Schematic representation of the OPA1 protein delineating its various domains; the variant's position is marked by a black arrow.

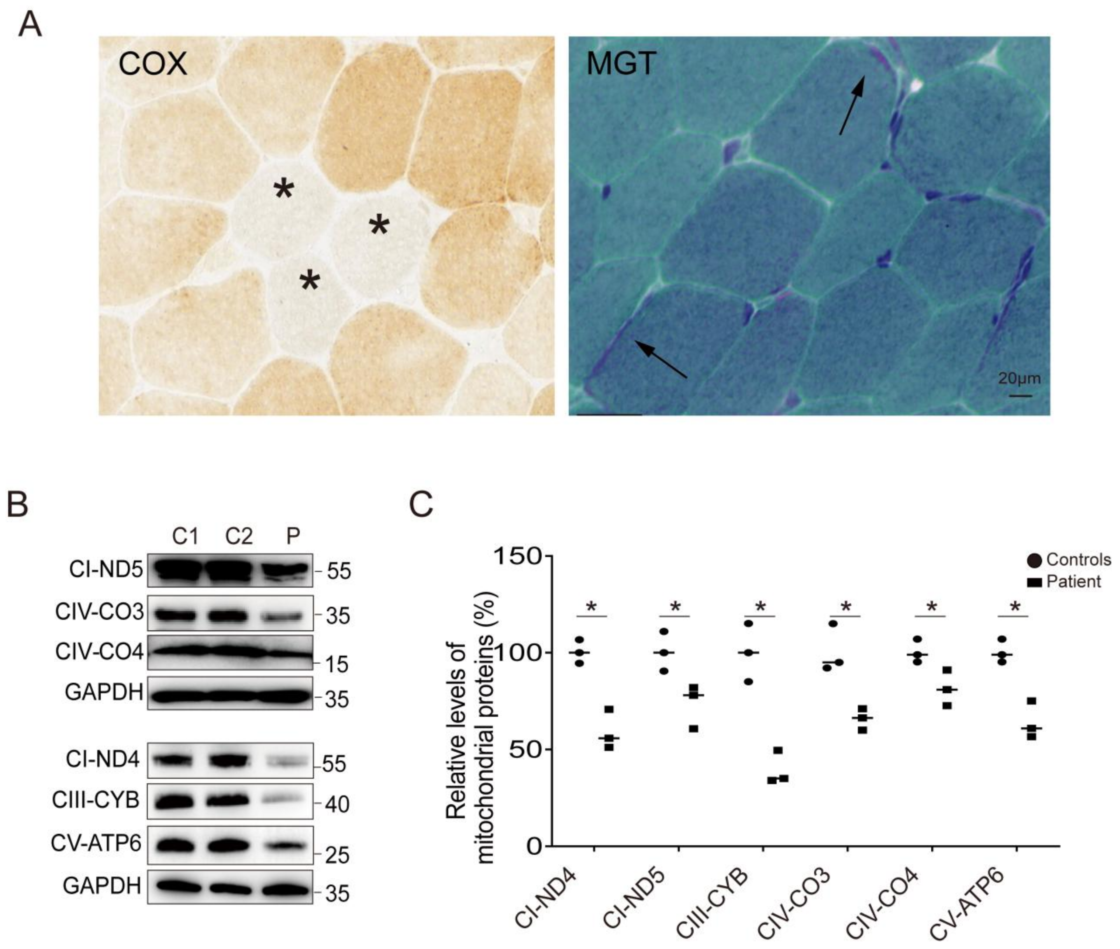

Supplementary Figure 2. Pathological analysis of muscle from the patient and comparative analysis of mitochondrial protein levels in muscle samples. (A) The muscle pathology of the patient indicates COX-deficient fibers in Cytochrome c Oxidase (COX) staining and subsarcolemmal accumulation as highlighted by modified gomori trichrome (MGT) staining. **(B)** Western blot analysis illustrated the levels of nDNA-encoded mitochondrial respiratory chain complex subunits (ATP5A, UQCRC2, CO4, SDHB, NDUFB8) and mtDNA-encoded subunits (ATP6, CO2, CO3, ND4, ND5, CYB). ND5,CO3,CO4 are derived from the same samples run on different but concurrent blots. ND4,CYB,ATP6 are derived from the same samples run on different but concurrent blots. **(C)** Densitometric quantification of the indicated mitochondrial proteins was normalized to GAPDH and is expressed as a percentage relative to the control group. Statistical analysis was by unpaired, 2-tailed t test, $*P < 0.05$.

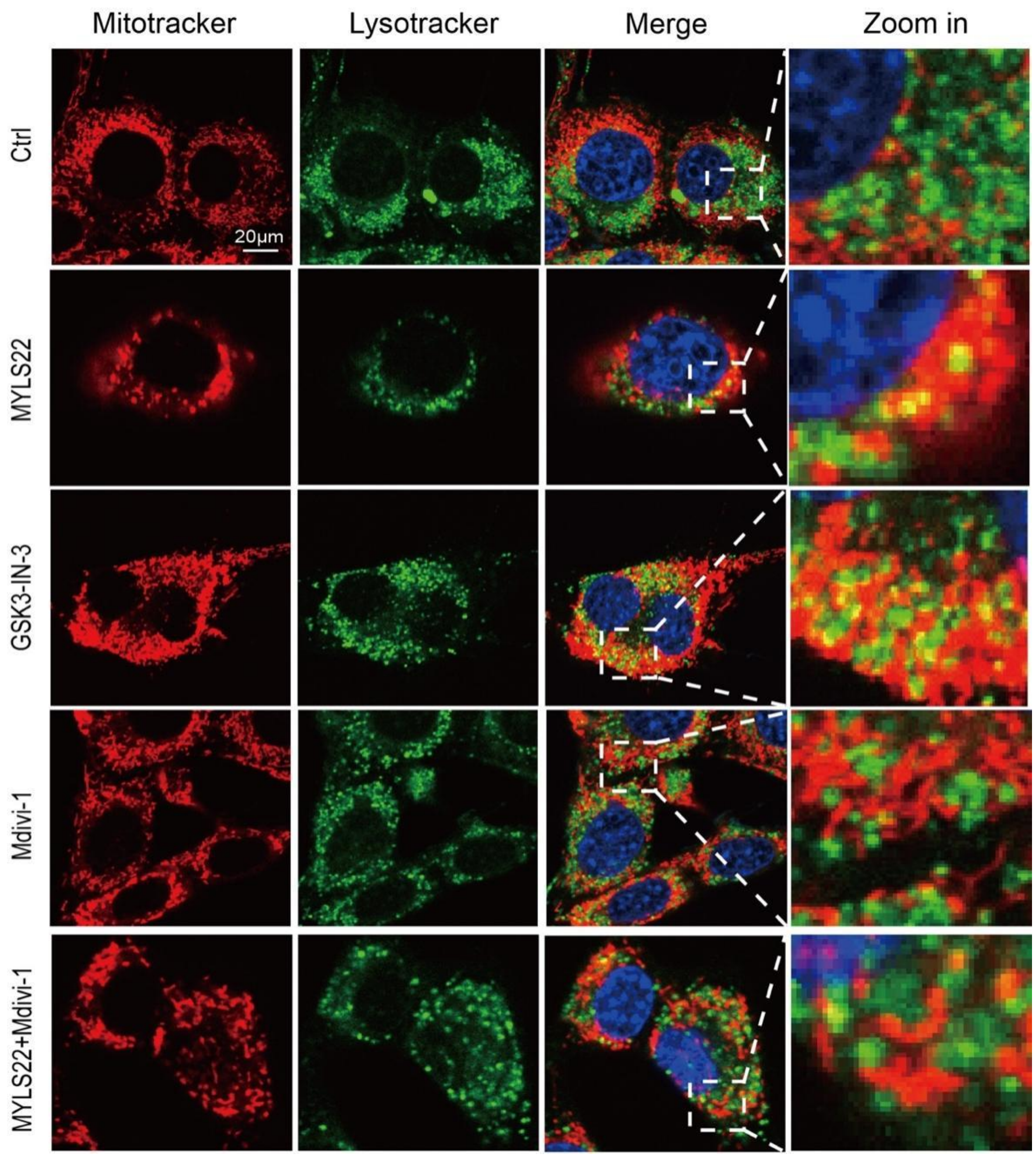

Supplementary Figure 3. Confocal microscopy images of cellular mitochondrial and lysosomal distribution under different pharmacological treatments.

Confocal microscopy images of MitoTracker (red) and LysoTracker (green) stained cells. The applied treatments include a control, MYLS22, GSK3-IN-3, Mdivi-1, and a combination of MYLS22 and Mdivi-1. Merged images illustrate the colocalization of mitochondria and lysosomes.

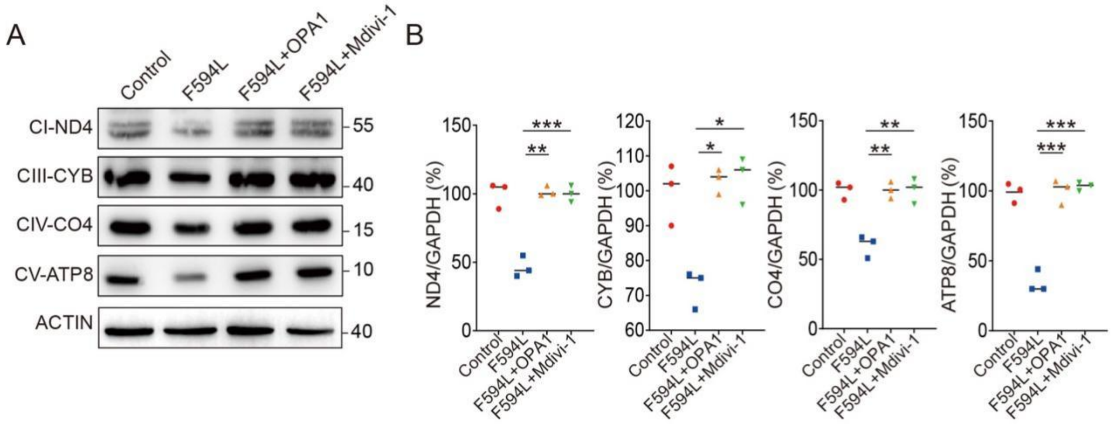

Supplementary Figure 4. Western blotting to evaluate the levels of other respiratory chain complex subunits. (A) Western blot analysis of mitochondrial respiratory chain complexes, including CI-ND4, CIII-CYB, CIV-CO4, and CV-ATP8. The results are derived from the same samples run on different but concurrent blots. **(B)** Data quantification indicates the effects of OPA1 overexpression and Mdivi-1 treatment on complex expression levels. Statistical analysis was by 1-way ANOVA and Tukey’ s post hoc test, *P < 0.05; **P < 0.01; ***P < 0.001 (B).

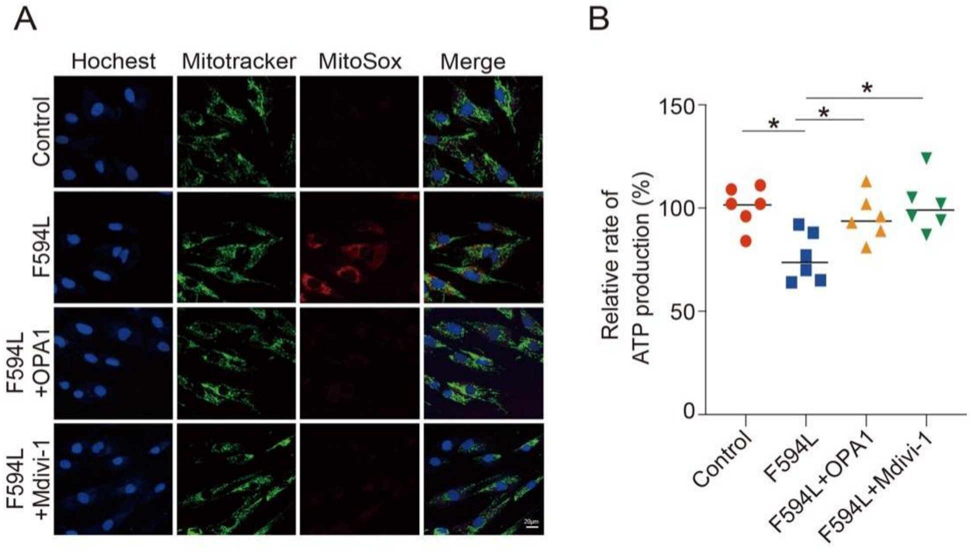

Supplementary Figure 5: Mdivi-1 mitigates ROS production and ATP synthesis impairment in F594L mutant cells. (A) Fluorescence microscopy of mitochondrial ROS in control, F594L mutant, F594L+OPA1, and F594L+Mdivi-1 treated fibroblasts. Mitochondria were stained with MitoTracker (green), and oxidative stress by MitoSOX (red). Hoechst (blue) stained the nucleus. **(B)** Measurement of the

relative ATP production rate in the various groups; the bar graph shows the proportion of ATP production compared to the control group. Statistical analysis was by 1-way ANOVA and Tukey’ s post hoc test, *P < 0.05 (B).
