# Supplementary material for: Targeting DRP1 with Mdivi-1 to correct mitochondrial abnormalities in ADOA+ syndrome
Source: JCI Insight. 2024 Jun 25;9(15):e180582. doi: 10.1172/jci.insight.180582 (PMC11383607; doi:10.1172/jci.insight.180582)

Full unedited gel for Figure 1D

OPA1

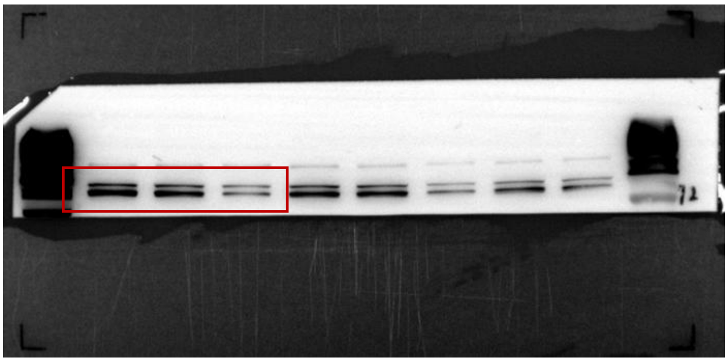

ACTIN

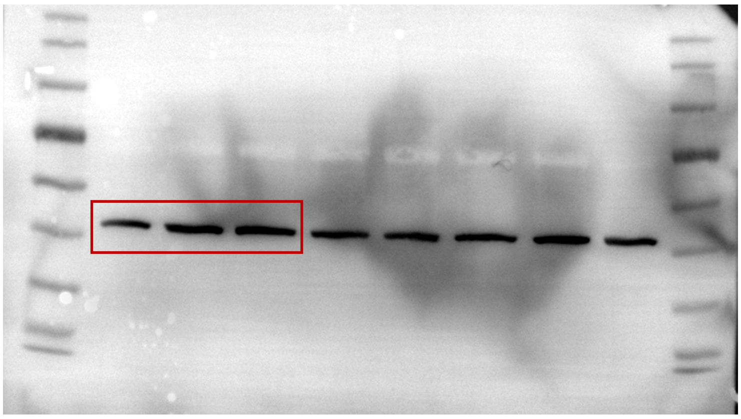

Full unedited gel for Figure 1G

ATP5A UQCRC2 SDHB CO2 NDUFB8

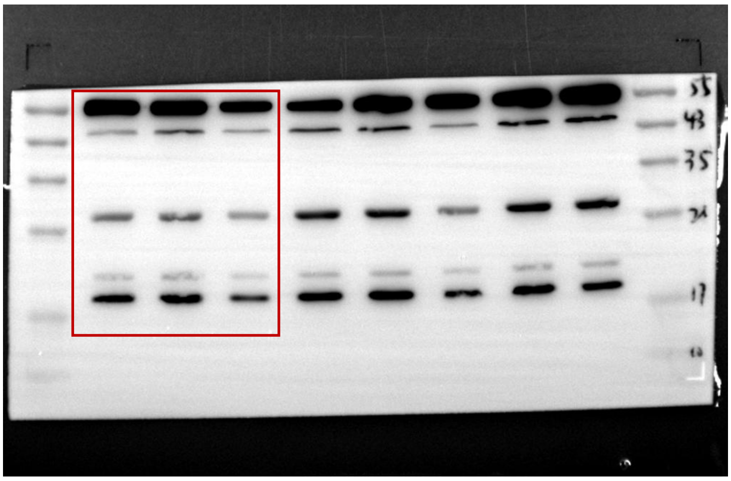

ACTIN

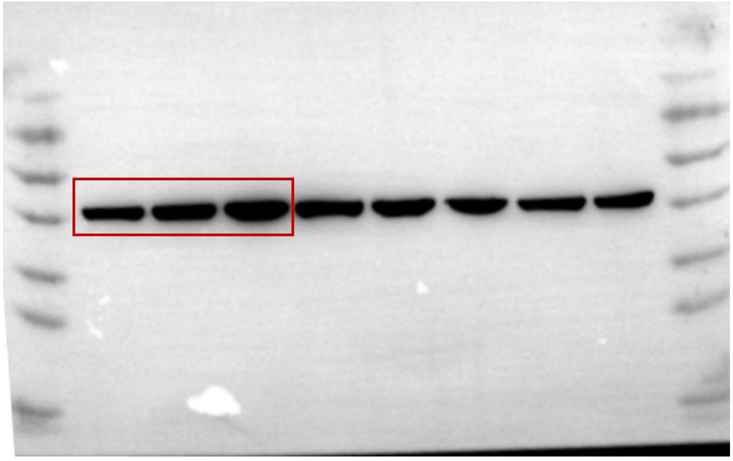

Full unedited gel for Figure 2

ND2

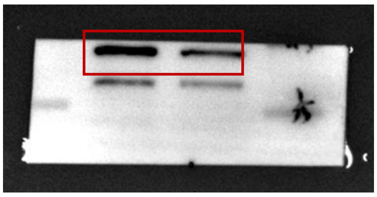

CYB

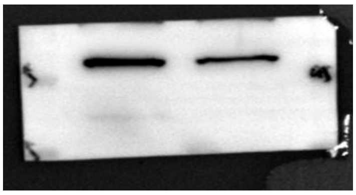

CO4

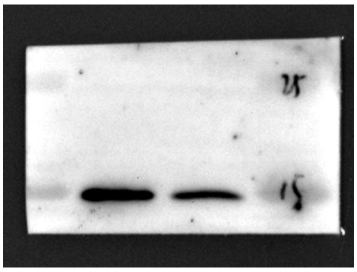

ACTIN

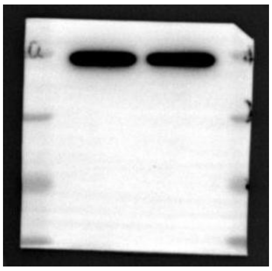

Full unedited gel for Figure 3B

OPA1

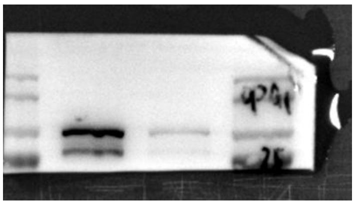

MFN1

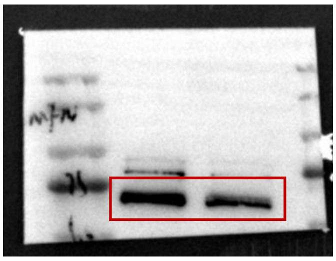

ACTIN

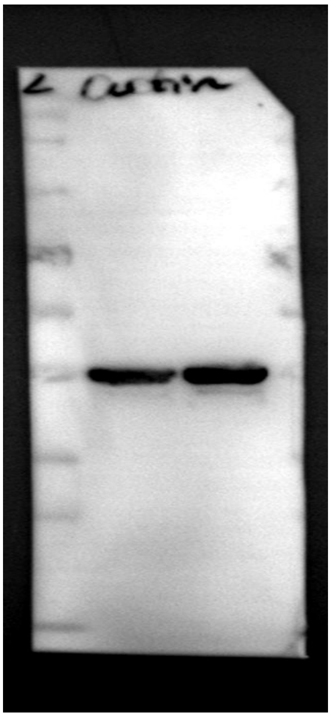

DRP1

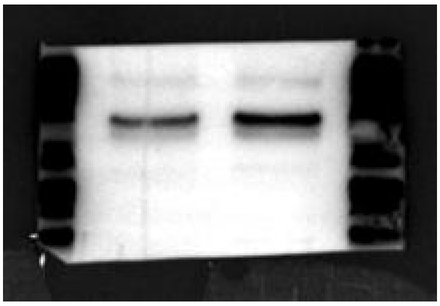

p-DRP1

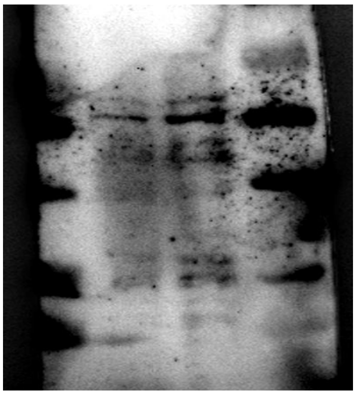

Full unedited gel for Figure 3C

DRP1

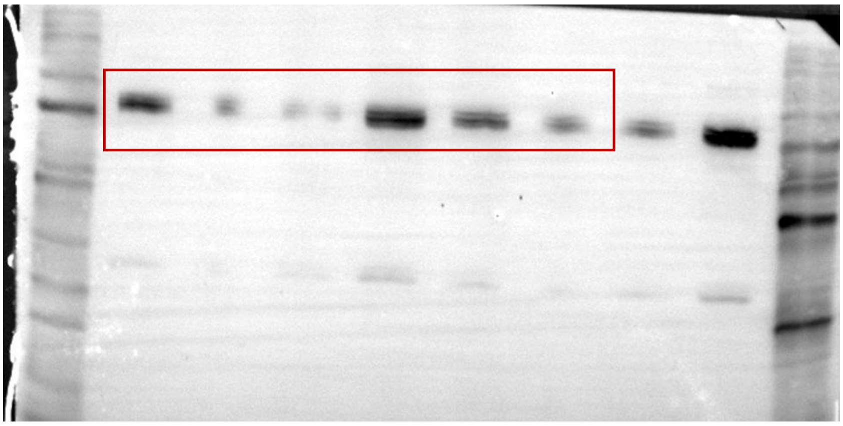

VDAC1

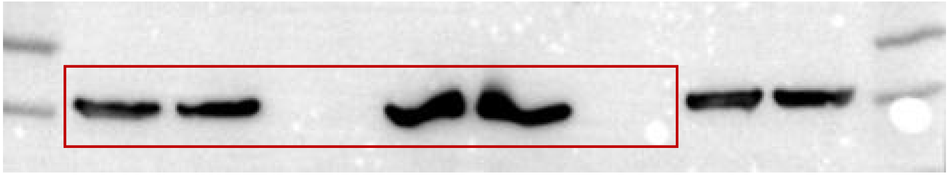

P-DRP1

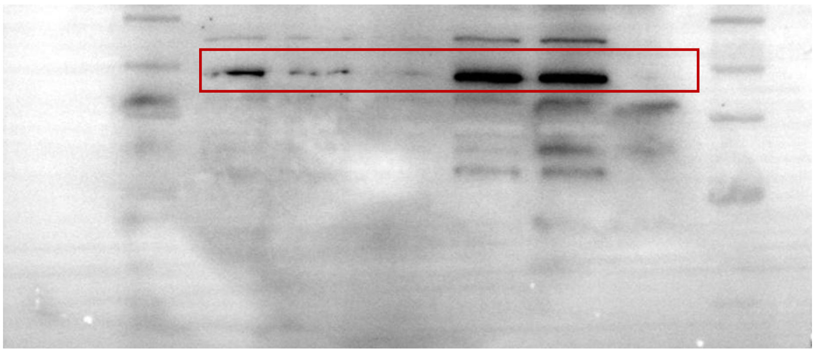

VDAC1

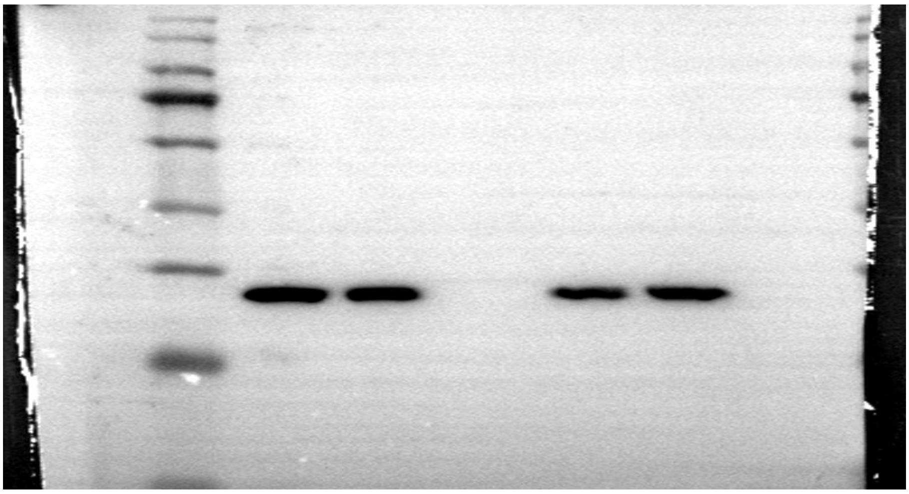

Full unedited gel for Figure 4A

LAMP1

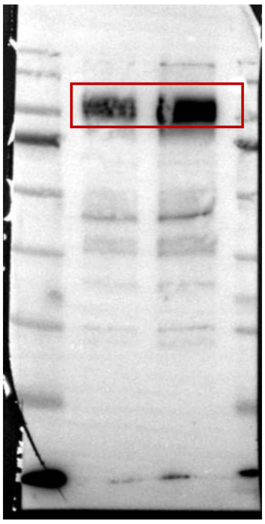

P62

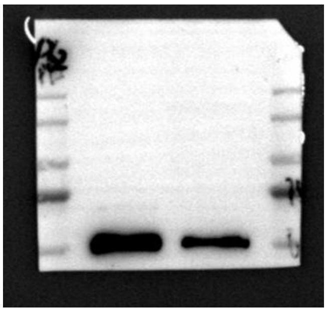

ACTIN

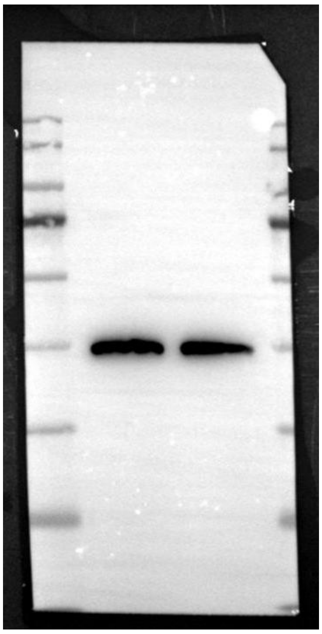

LC3

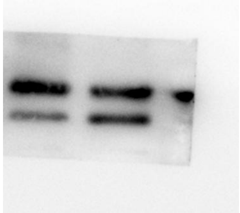

ACTIN

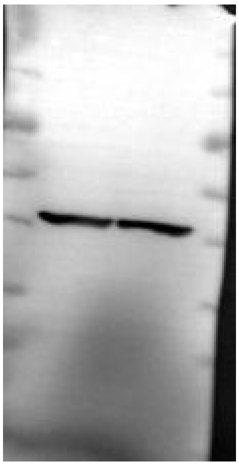

Full unedited gel for Figure 4B

PINK1

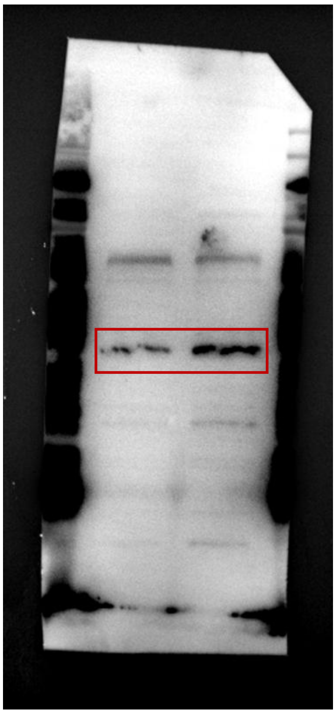

BNIP3L

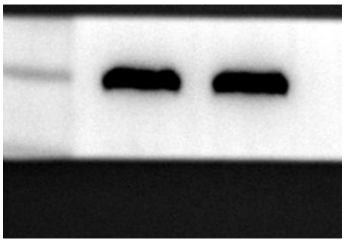

ACTIN

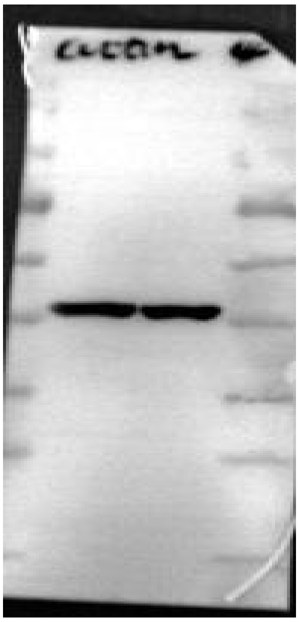

FUNDC1

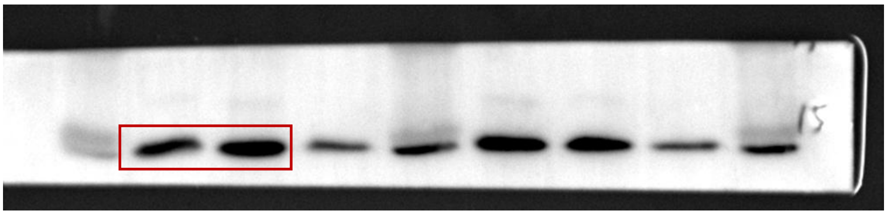

ACTIN

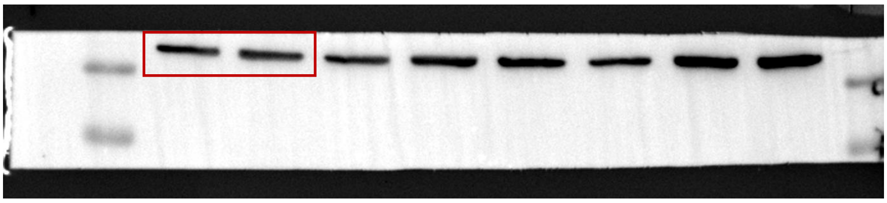

Full unedited gel for Figure 5A

OPA1

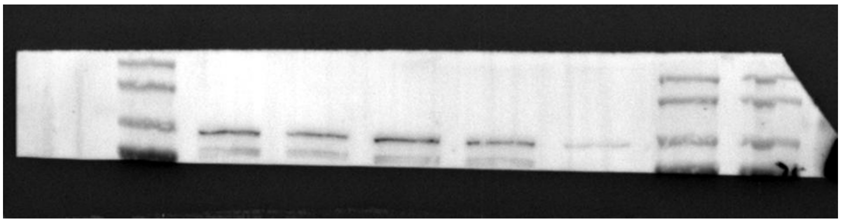

ACTIN

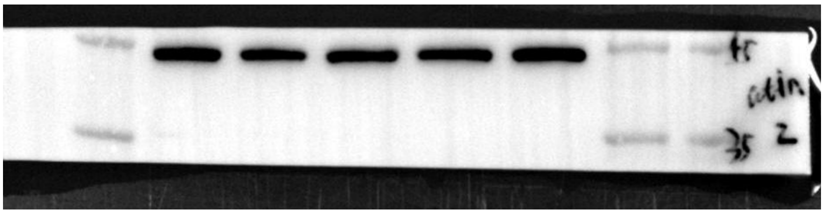

DRP1

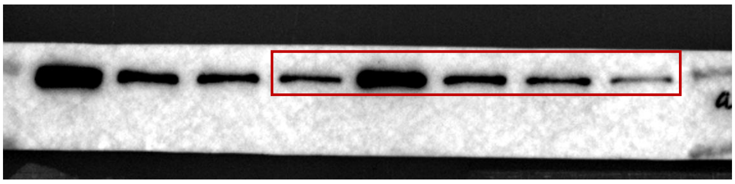

P-DRP1

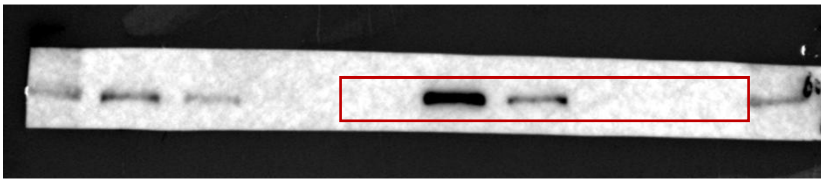

ACTIN

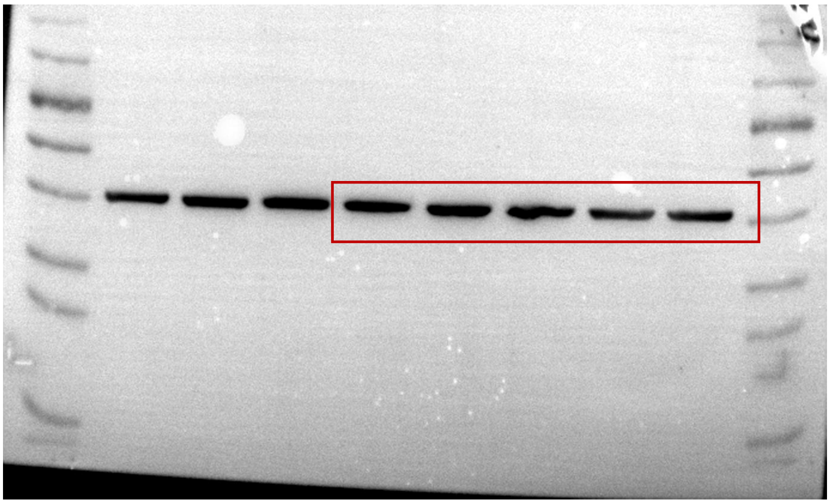

Full unedited gel for Figure 5D

OPA1

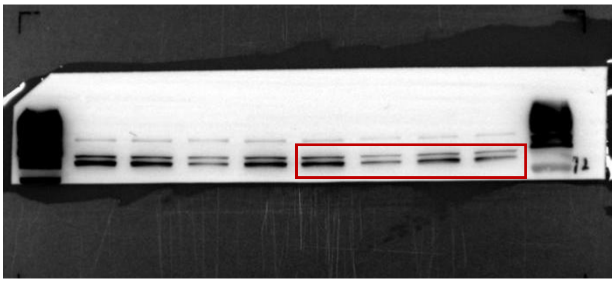

ACTIN

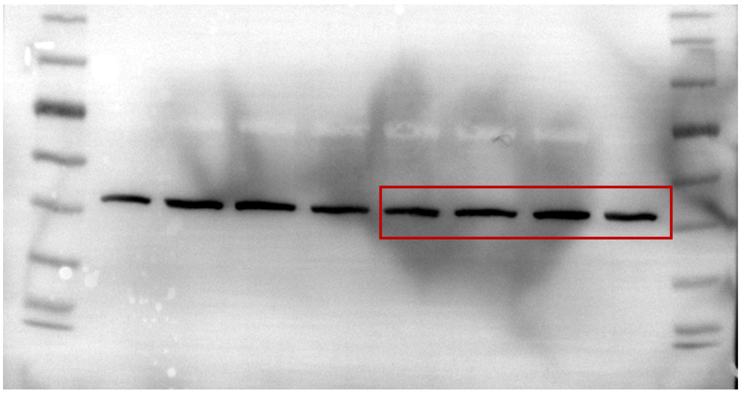

LC3

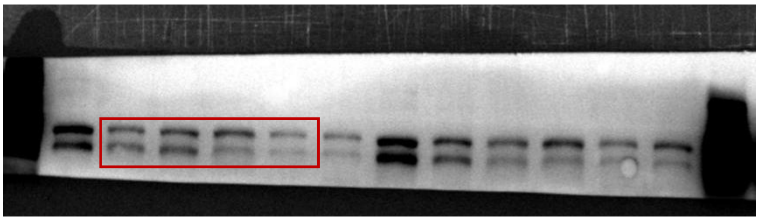

ACTIN

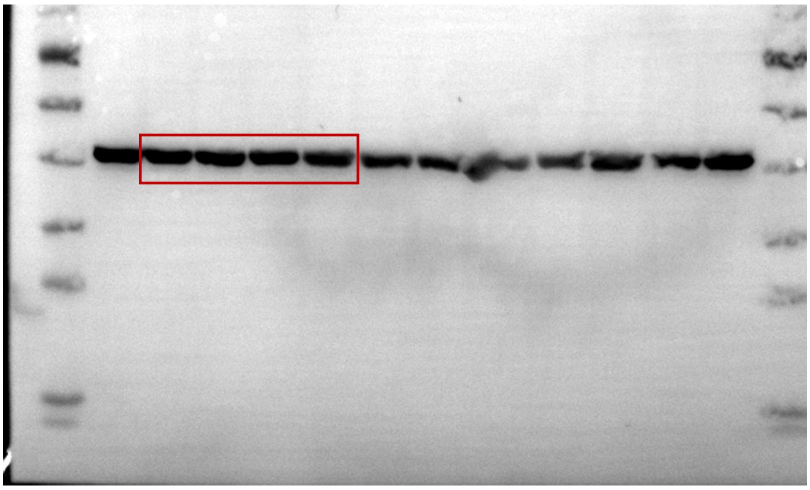

Full unedited gel for Figure 5E

ATP5A UQCRC2 SDHB CO2 NDUFB8

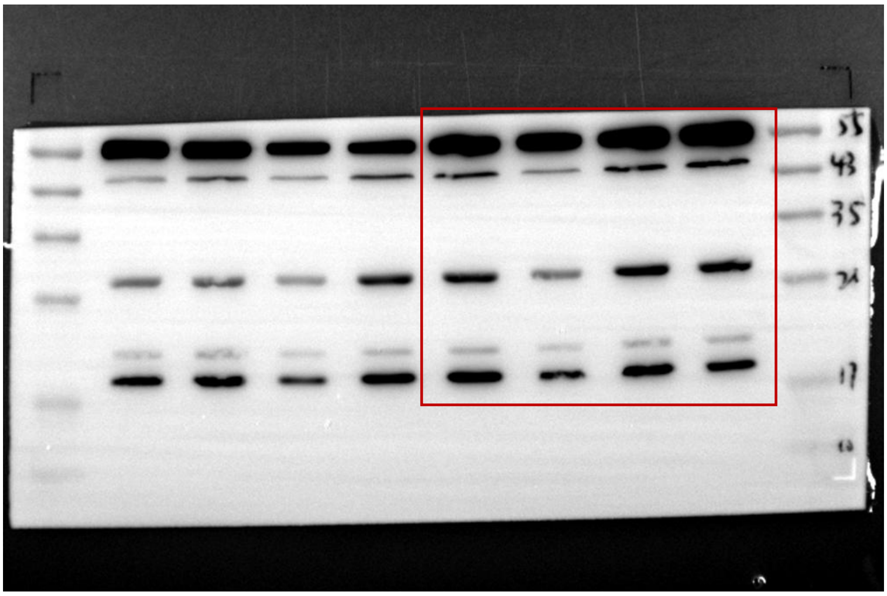

ACTIN

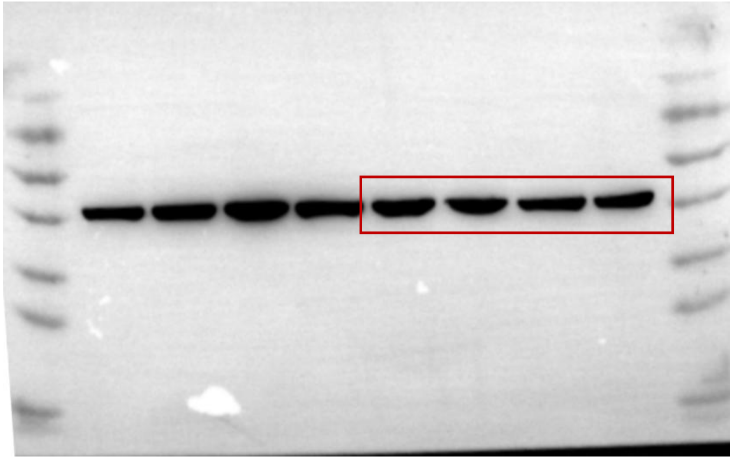

Full unedited gel for supplement Figure 2A

ND5

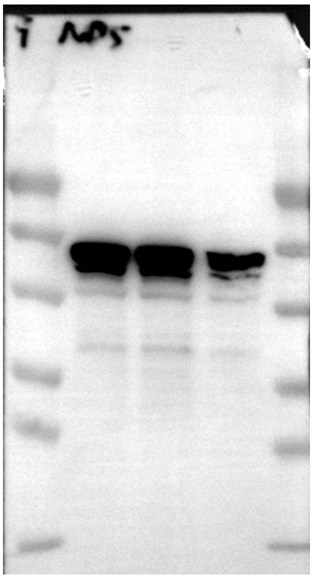

CO3

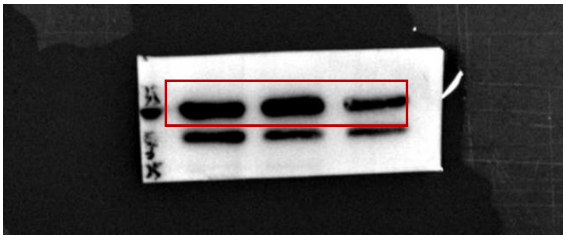

CO4

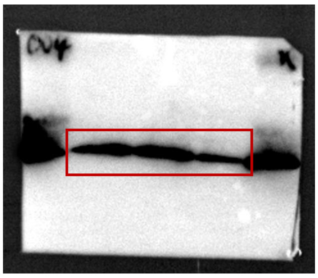

GAPDH

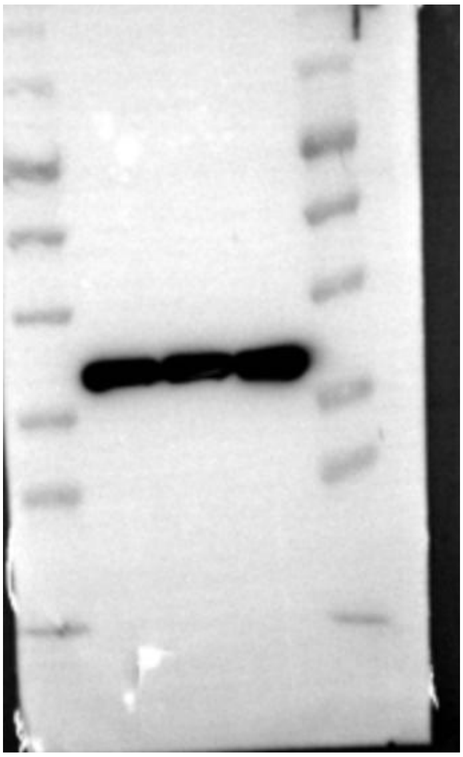

ND4

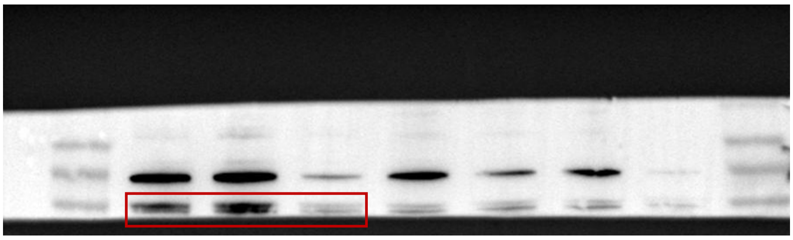

CYB

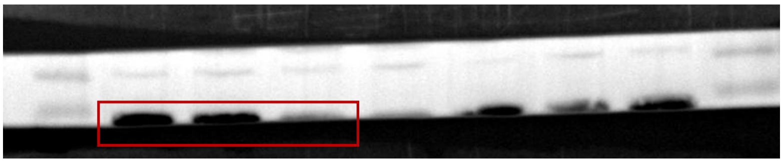

ATP6

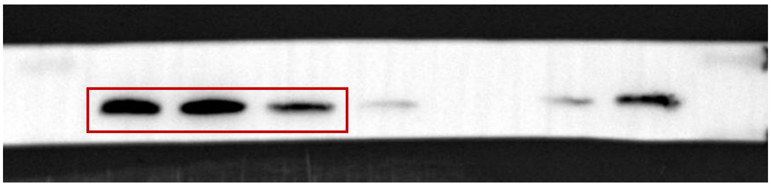

GAPDH

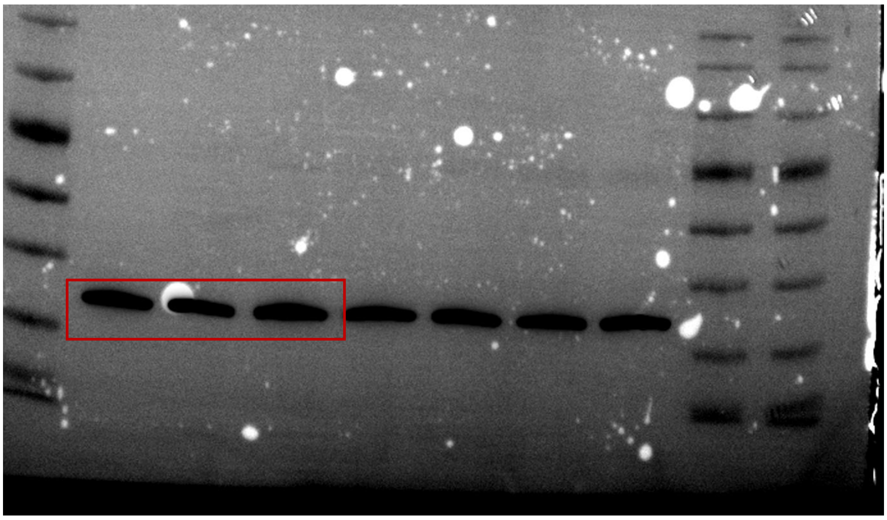

Full unedited gel for supplement Figure 4A

ND4

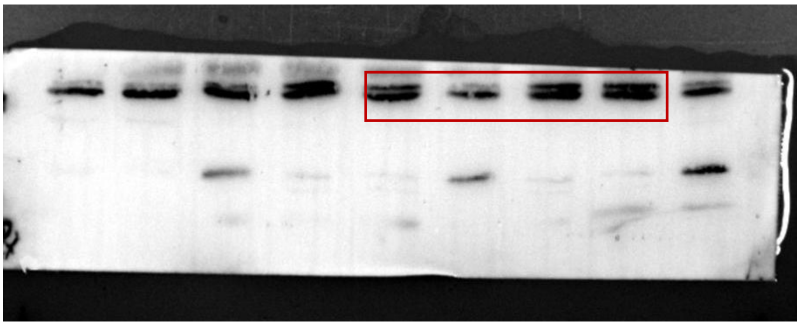

CYB

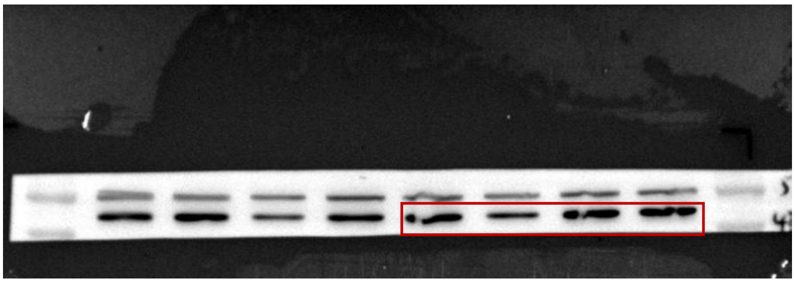

CO4

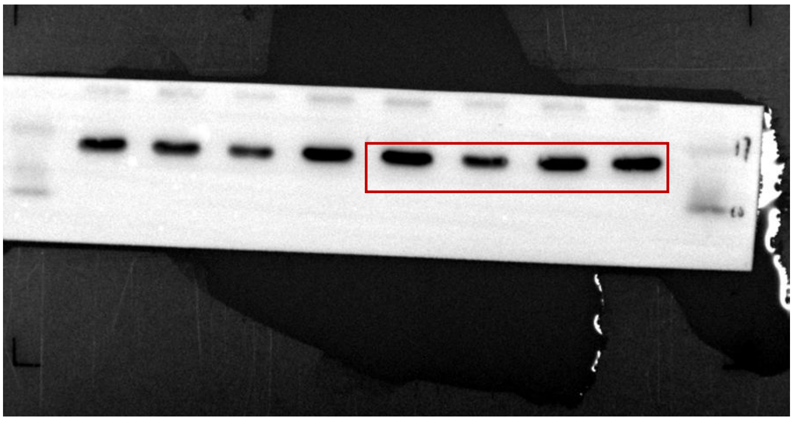

ATP8

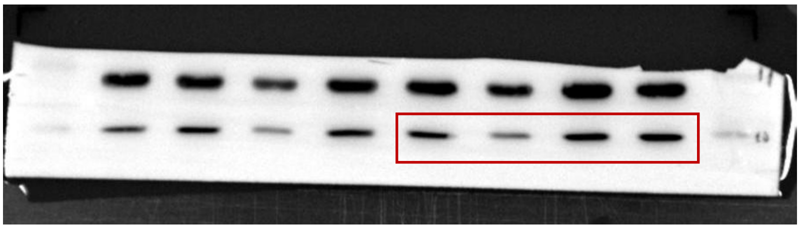

ACTIN

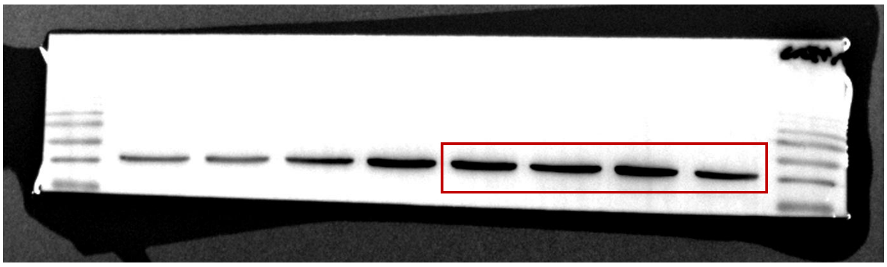

Supplement: Unedited blot and gel images [file jciinsight-9-180582-s135.pdf]
